# Immobilized IgG-containing immune complexes require platelets to recruit neutrophils during inflammation

**DOI:** 10.1172/JCI195987

**Published:** 2025-12-02

**Authors:** Marie Bellio, Isabelle Allaeys, Etienne Doré, Myriam Vaillancourt, Tania Lévesque, Mélina Monteil, Nicolas Vallières, Philippe Desaulniers, Nicolas Bertrand, Valance A. Washington, Yotis Senis, Steve Lacroix, Paul Fortin, Clémence Belleannée, Eric Boilard

**Affiliations:** 1 Faculté de Médecine de l’Université Laval, Université Laval, Québec City, Québec, Canada.; 2 Centre de Recherche ARThrite – Arthrite, Recherche, Traitements, Université Laval, Québec City, Québec, Canada.; 3 Axe maladies infectieuses et immunitaires du Centre de recherche du Centre hospitalier universitaire de Québec-Université Laval, Québec City, Québec, Canada.; 4 Axe neurosciences du Centre de Recherche du Centre Hospitalier Universitaire de Québec-Université Laval et Département de Médecine Moléculaire de l’Université Laval, Québec City, Québec, Canada.; 5 Axe Endocrinologie et Néphrologie du Centre de Recherche du Centre Hospitalier Universitaire de Québec – Université Laval, Québec City, Québec, Canada.; 6 Faculté de Pharmacie de l’Université Laval, Québec City, Québec, Canada.; 7 Oakland University, Department of Biological Sciences, Rochester, Michigan, USA.; 8 Centre for Cardiovascular and Nutrition Research, Institut National de la Santé et de la Recherche, Faculté de Médicine, Aix-Marseille University, Marseille, France.; 9 Centre de Recherche en Reproduction, Développement et Santé Intergénérationnelle – Université Laval, Québec City, Québec, Canada.; 10 Axe Reproduction du Centre de Recherche du Centre Hospitalier Universitaire de Québec – Université Laval, Québec City, Québec, Canada.

**Keywords:** Autoimmunity, Immunology, Arthritis, Neutrophils, Platelets

## Abstract

During vascular injury, platelets are essential for halting bleeding and recruiting neutrophils to prevent microbial invasion. However, in antibody-mediated autoimmune diseases occurring without vascular damage, neutrophils infiltrate tissues and contribute to pathology. Here, we investigated whether the dependence of neutrophils on platelets is conserved in the context of antibody-driven inflammation. Using human cells from individuals with rheumatoid arthritis and a microfluidic system mimicking physiological shear over IgG-containing immune complexes, we demonstrate that despite expressing Fc receptors, neutrophils required platelets to stably adhere to immune complexes under flow. Platelet Fcγ receptor 2a (FcγRIIA) binding was critical for resisting shear stress, while neutrophils used FcγRIIA and FcγRIIIB for immune complex recognition. Platelet P-selectin binding to neutrophil P-selectin glycoprotein ligand 1 (PSGL-1) was essential for recruitment, whereas macrophage-1 antigen (Mac-1) was dispensable. In a mouse model of autoantibody-mediated arthritis, intravital imaging confirmed that neutrophil recruitment relied on PSGL-1. Importantly, expression of FcγRIIA aggravated arthritis, and blockade of PSGL-1, but not Mac-1, in these mice abrogated both the platelet and neutrophil interactions and disease. These findings identify key molecular interactions in platelet-neutrophil cooperation and reveal that platelets are essential enablers of FcR-mediated neutrophil adhesion in antibody-driven inflammation.

## Abstract 2

Neutrophils migrate in tissue to play a pathogenic role in autoimmune arthritis. This study shows that they fail to migrate in the absence of platelets.

## Graphical abstract

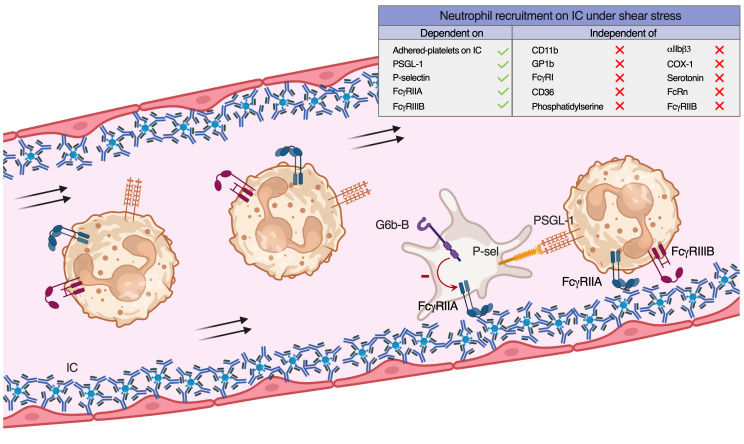

## Introduction

Antigen-antibody molecular scaffolds, called immune complexes (ICs), participate in opsonization, phagocytosis, and cellular activation by receptors belonging to the Fcγ receptor (FcγR) family (1–3). ICs can arise during sepsis or viremia when the infected host has developed immunity against microbial antigens or toxins (4, 5). In autoimmune rheumatic diseases, antibodies recognize self-antigens, forming ICs that deposit on blood vessel walls and tissues (6–12). IC deposition plays a pathogenic role in autoimmune diseases, and mice lacking activating FcγRs (due to the absence of the Fc common γ chain) are protected in lupus nephritis and rheumatoid arthritis (RA) models (13–15).

Neutrophils, the most abundant leukocytes in human blood circulation, are commonly recognized as the primary responders to immune triggers. Human neutrophils constitutively express 2 low-affinity FcγRs — FcγRIIA and FcγRIIIB — in addition to FcγRI, which is inducible (2, 16). It is notable that the FcγRs in mice differ from those in humans. Mice do not express FcγRIIA, and murine neutrophils express FcγRIII and FcγRIV (2). Neutrophils can contribute to pathogenesis by releasing a diverse proinflammatory arsenal, comprising granule content (proteases), ROS, and lipid mediators, as well as by undergoing NETosis, which can be induced through FcγR signaling (17).

Considering the prevalence of neutrophils in the bloodstream, specific signals must orchestrate swift recruitment of these neutrophils to affected tissues. Traditional models of neutrophil adhesion to the blood vessel wall involve (a) rolling along the activated endothelium through selectin binding, followed by (b) firm adhesion involving macrophage-1 antigen (Mac-1) (CD11b/CD18) integrins and ICAM-1 on endothelial cells (18–20). However, investigations into neutrophil recruitment in autoimmune diseases involving ICs have unveiled alternative mechanisms contributing to pathogenesis. Utilizing microfluidic systems to replicate intravital shear stress and in vivo models of IC-mediated nephritis, research has highlighted that FcγRIIIB, strategically localized in the microvilli of neutrophil membranes to enhance substrate recognition, is pivotal for initial tethering on the IC surface and slow rolling (21). FcγRIIA plays a nonredundant role in neutrophil recruitment (22, 23), and the presence of shear force augments F-actin polymerization, fostering robust FcγRIIA catch bonds with IgG (i.e., bonds with interactions that are paradoxically prolonged by force) (24, 25). Of note, these models suggest that Mac-1 integrins are necessary for both FcγRIIIB and FcγRIIA to sustain neutrophil adhesive functions on ICs (21, 26–29).

Unlike leukocytes, which can roll on the activated endothelium, the majority of platelets position themselves as flat sliding discs on the damaged vessel wall, which may form a thrombus (30, 31). This positioning allows platelets to maximize coverage of the wound surface and, given their small dimensions and absence of a nucleus, potentially minimizes the drag forces induced by heightened shear stress near the vascular wall (31). Given that nearly 1 trillion platelets circulate in the bloodstream, their specialized features, including their capability to form membrane protrusions (32), significantly bolster their ability to maintain vascular integrity and prevent bleeding (31).

Intriguingly, platelet studies conducted over 100 years ago uncovered instances of interactions between leukocytes and platelet plugs (33). Studies have shown that, in the absence of platelets, neutrophils display impaired migration and aberrant crawling behavior in response to inflammatory stimuli such as cytokines (34, 35). While the interaction between platelets and neutrophils can be mediated by P-selectin binding to P-selectin glycoprotein ligand 1 (PSGL-1) (34, 36), numerous additional ligand-receptor pairs have also been identified. For instance, platelet integrin αIIbβ3, glycoprotein Ib (GPIb) or junctional adhesion molecule 3 (JAM-3) can bind to Mac-1 on neutrophils (29, 37–40). Activated αIIbβ3 can also bind SLC44A2 on neutrophils, supporting flow-dependent NETosis (41). Moreover, under thromboinflammatory conditions, platelet activation leads to the generation of procoagulant platelets, which expose phosphatidylserine (PS) — a ligand that can interact with CD36 on neutrophils (42).

Although neutrophils express the necessary FcγRs to directly bind ICs, it remains unknown whether platelets similarly support neutrophil recruitment in conditions involving IC deposition. This knowledge gap is largely due to the absence of FcγRs on mouse platelets, which limits the ability to model and delineate platelet functions in antibody-mediated diseases. This stands in contrast to human platelets, which express FcγRIIA and are thus more likely to participate directly in IC-driven inflammation. These species differences underscore a critical need for further investigation into the role of platelets in IC-mediated pathology, particularly using models that more accurately reflect human platelet biology.

In this context, we hypothesized that platelets could play a role in neutrophil recruitment by ICs in inflammatory conditions. To explore this hypothesis, we investigated molecules known to be involved in platelet and neutrophil interactions in hemostasis and whether the functions mediated by these molecules are conserved in immune contexts involving ICs.

## Results

**Figure 1:**
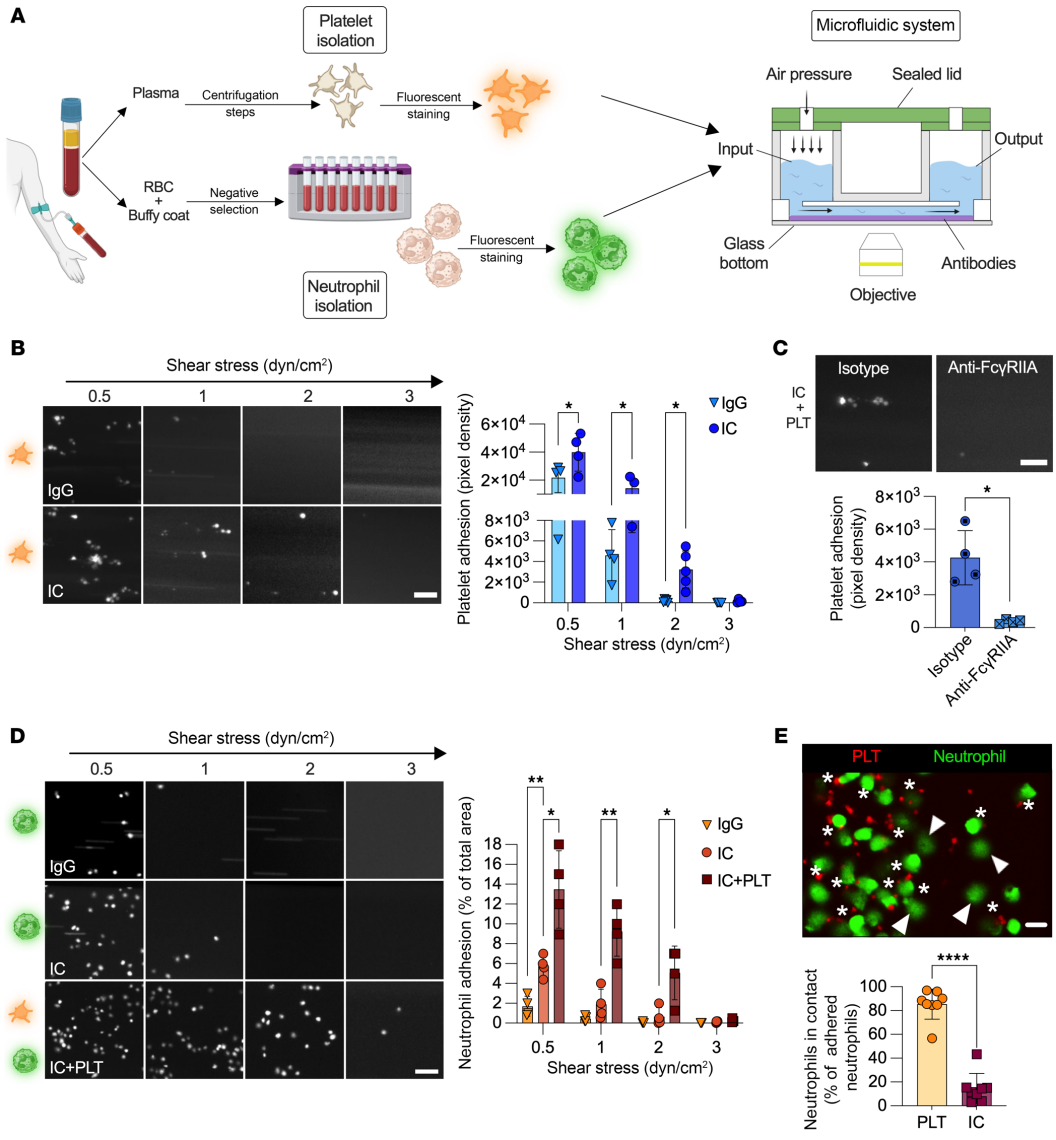
Human platelets are essential for neutrophil adhesion to ICs under shear stress. (**A**) Experimental design schematic: platelets and neutrophils from blood were isolated and stained red blood cells (RBC). A microfluidic approach was used to evaluate platelet-neutrophil interactions mediated under flow conditions. Platelets were perfused in microcapillaries coated with monomeric IgGs and ICs, followed by neutrophils. (**B**) Platelet adhesion on surfaces coated with IgG and IC at different shear stresses. Representative images (30 minutes, scale bar: 10 μm) and platelet adhesion quantification are shown. *P < 0.05, by 2-way ANOVA with uncorrected Fisher’s LSD (*n* = 4–5). (**C**) Platelets (PLT) incubated or not (isotype) with anti-FcγRIIA antibody perfused in microcapillaries IC-coated at 2 dyn/cm^2^. Representative images (30 minutes; scale bar: 10 μm) and platelet adhesion quantification are shown. *P < 0.05, by paired *t* test (*n* = 5). (**D**) Neutrophil adhesion on surface coated with IgG, ICs, or ICs plus platelets at different shear stresses. Representative images (15 minutes, scale bar: 50 μm) and neutrophil adhesion quantification are shown. *P < 0.05 and **P < 0.01, by 2-way ANOVA with uncorrected Fisher’s least-squares difference (LSD) (*n* = 4). (**E**) Representative image of adherent neutrophils on ICs in the presence of platelets (scale bar: 10 μm). Arrowheads indicate some neutrophils only in contact with ICs (without platelet contact), and asterisks indicate some neutrophils in contact with platelets. The percentage of neutrophils adhered on platelets or on ICs (without platelet contact) was quantified. ****P < 0.0001, by unpaired *t* test (*n* = 8). Data are presented as the mean ± SD.

Human platelets were introduced into a microfluidic system, where various shear stresses were applied to assess the binding of washed platelets to ICs. A potential confounding factor is that both platelets and neutrophils express numerous receptors capable of binding pathogen- or damage-associated molecular patterns (43). Thus, to focus specifically on binding to FcγRs rather than antigens, we used heat-aggregated IgG as a surrogate for ICs (4, 44). The majority of these aggregates had diameters of 160 nm (4). We found that human platelets promptly halted and accumulated on ICs and that this occurred most efficiently under conditions of shear stress of 2 dyn/cm^2^ and lower. The blockade of FcγRIIA with monoclonal anti-FcγRIIA–blocking antibodies completely abrogated the interaction, consistent with prior studies showing that this receptor is required for IC-platelet interactions (45, 46). Moreover, platelets failed to adhere on monomeric IgG in conditions of shear stress, confirming the role of FcγRIIA in the specific recognition of ICs (Figure 1, A–C, and Supplemental Figure 1; supplemental material available online with this article; https://doi.org/10.1172/JCI195987DS1). Human neutrophils, isolated by a negative selection approach to ensure they did not include significant numbers of platelets (less than 1 platelet per 1,000 neutrophils), were injected in the same system, in the presence or absence of platelets and ICs. In shear stress of 1 dyn/cm^2^ and higher, we found that neutrophils did not adhere to ICs and were washed away unless platelets were present (Figure 1D, Supplemental Figure 1, and Supplemental Videos 1 and 2). Moreover, they failed to efficiently bind to monomeric IgG (Figure 1D). We thus used the above experimental conditions (2 dyn/cm^2^), consistent with shear stresses measured in microvessels in inflamed organs or tissues (19, 21, 47), and given the specificity of the measured cells’ binding to ICs relatively to monomeric IgG, to determine how platelets recruit neutrophils on deposited ICs. Using fluorescence labeling to distinguish platelets and neutrophils, we determined that 85.4% of the neutrophils colocalized with platelets (Figure 1E). The addition of erythrocytes to the in vitro system did not enhance neutrophil interactions with ICs, indicating that the strong dependence on platelets was not simply due to a lack of erythrocyte-driven displacement of neutrophils toward the marginal zone (Supplemental Figure 1). Thus, although human neutrophils express activating FcγRs, they must interact with platelets in order to immobilize on deposited ICs under physiological flow conditions.

**Figure 2:**
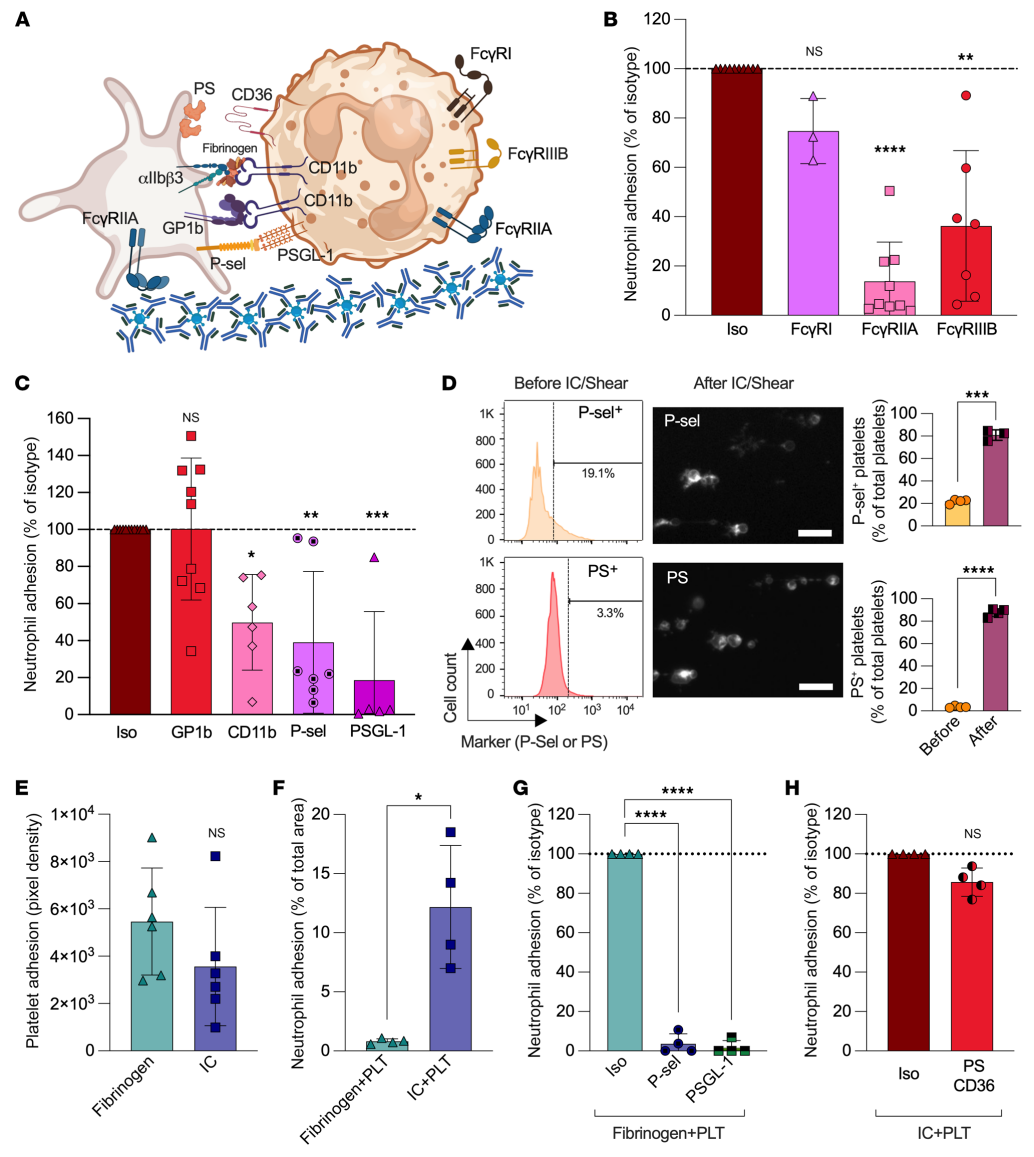
Dissecting the interactions between human platelets and neutrophils in the presence of ICs. Platelets and neutrophils were isolated from healthy volunteers and perfused in microcapillaries at 2 dyn/cm^2^. (**A**) Schematic of molecules involved in platelet-neutrophil adhesion. (**B** and **C**) Platelets were perfused in IC-coated microcapillaries. (**B**) Neutrophils were perfused in the presence of a control (Iso) or blocking antibody against Fc receptors. (**C**) Adhered platelets were incubated with blocking antibodies. Neutrophils were perfused in the presence of a control or blocking antibody as indicated. The area covered by neutrophils is quantified and expressed as a percentage of the isotype control. *P < 0.05, **P < 0.01, ***P < 0.001, and ****P < 0.0001, by Kruskal-Wallis test with Dunn’s multiple-comparison test (*n* = 3–9). (**D**) P-selectin (P-sel) and phosphatidylserine (PS) expression were evaluated on platelets before and after adhesion on ICs. Representative histograms (flow cytometry) and images (microfluidic 30 minutes; scale bars: 10 μm) are illustrated, and the percentage of platelets expressing P-selectin or PS was quantified. ***P < 0.001 and ****P < 0.0001, by unpaired *t* test with Welch’s correction (*n* = 3–4). (**E**–**H**) Platelets were perfused in microcapillaries coated with IC or fibrinogen, as indicated, followed by neutrophils. Platelet (**E**) and neutrophil (**F**) adhesion was measured. *P < 0.05, by Mann-Whitney *U* test (*n* = 4). (**G**) Platelets adhered on fibrinogen were incubated with an isotype or an antibody against P-selectin followed by neutrophil incubated with an isotype or a blocking antibody against PSGL-1 or P-selectin. ****P < 0.0001, by 1-way ANOVA with Dunnett’s test (*n* = 4). (**H**) Platelets adhered on ICs were incubated with an isotype or PS blocker followed by perfusion of neutrophils incubated with an isotype or PS blocker combined with an anti-CD36–blocking antibody. Mann-Whitney *U* test (*n* = 4). Data are presented as the mean ± SD.

To verify whether or not neutrophil FcγRs (FcγRI, FcγRIIA, and FcγRIIIB) were dispensable in this platelet-dependent process, we preincubated neutrophils with blockers of FcγRs prior to injection in the microfluidic system. While blockade of FcγRI showed no effects, the individual blockade of FcγRIIA (86.3%) and FcγRIIIB (63.8%) significantly inhibited neutrophil adhesion on ICs (Figure 2, A and B). Moreover, blockade of Mac-1, which is involved in the functions of both FcγRIIIB and FcγRIIA (21, 26–29), also significantly reduced neutrophil adhesion (Figure 2C). While these findings appear consistent with the respective nonredundant roles of FcγRIIIB and FcγRIIA in the tethering and formation of catch bonds (22, 24, 25), they are intriguing, as they also highlight an unrecognized role of platelets in these interactions.

The interactions between platelets and neutrophils can involve the specific binding of P-selectin and GPIb on platelets with PSGL-1 and Mac-1 on neutrophils (48–51). Thus, we interfered with these candidate molecules that might be involved in platelet-neutrophil interactions (Figure 2C and Supplemental Figure 2). Blockade of GPIb, using an antibody reportedly capable of interfering in vWF or Mac-1 binding (52, 53), had no effect. Individual blockade of PSGL-1 or its counterreceptor, P-selectin, reduced neutrophil adhesion measured at both 2 and 1 dyn/cm^2^ (Figure 2C and Supplemental Figure 3). The role of selectin was consistent with surface P-selectin on platelets, as it was promptly exposed by platelets bound to ICs (Figure 2D and Supplemental Figure 4). For comparison, we used fibrinogen as the immobilized surface, which was as potent as ICs at recruiting platelets (Figure 2E). Although very few neutrophils were recruited in the presence of platelets using immobilized fibrinogen compared with ICs (Figure 2F), blockade of P-selectin or PSGL-1 totally inhibited neutrophil adhesion in these FcγR-independent conditions (Figure 2G). During thromboinflammation, PS, expressed on procoagulant platelets, can further facilitate the binding of neutrophils to platelets immobilized on the vessel wall (42, 54). We thus examined its role in IC-mediated adhesion of neutrophils. PS was also induced on platelet surfaces upon contact with immobilized ICs (Figure 2D and Supplemental Figure 4), but was dispensable in the interactions with neutrophils, as the addition of annexin V or blockade of the PS counterreceptor CD36 had no effect (Figure 2H). Taken together, the data point to an intricate mechanism of cellular adhesion involving neutrophil FcγRs as well as molecules from platelets, possibly occurring in a chronological sequence.

**Figure 3:**
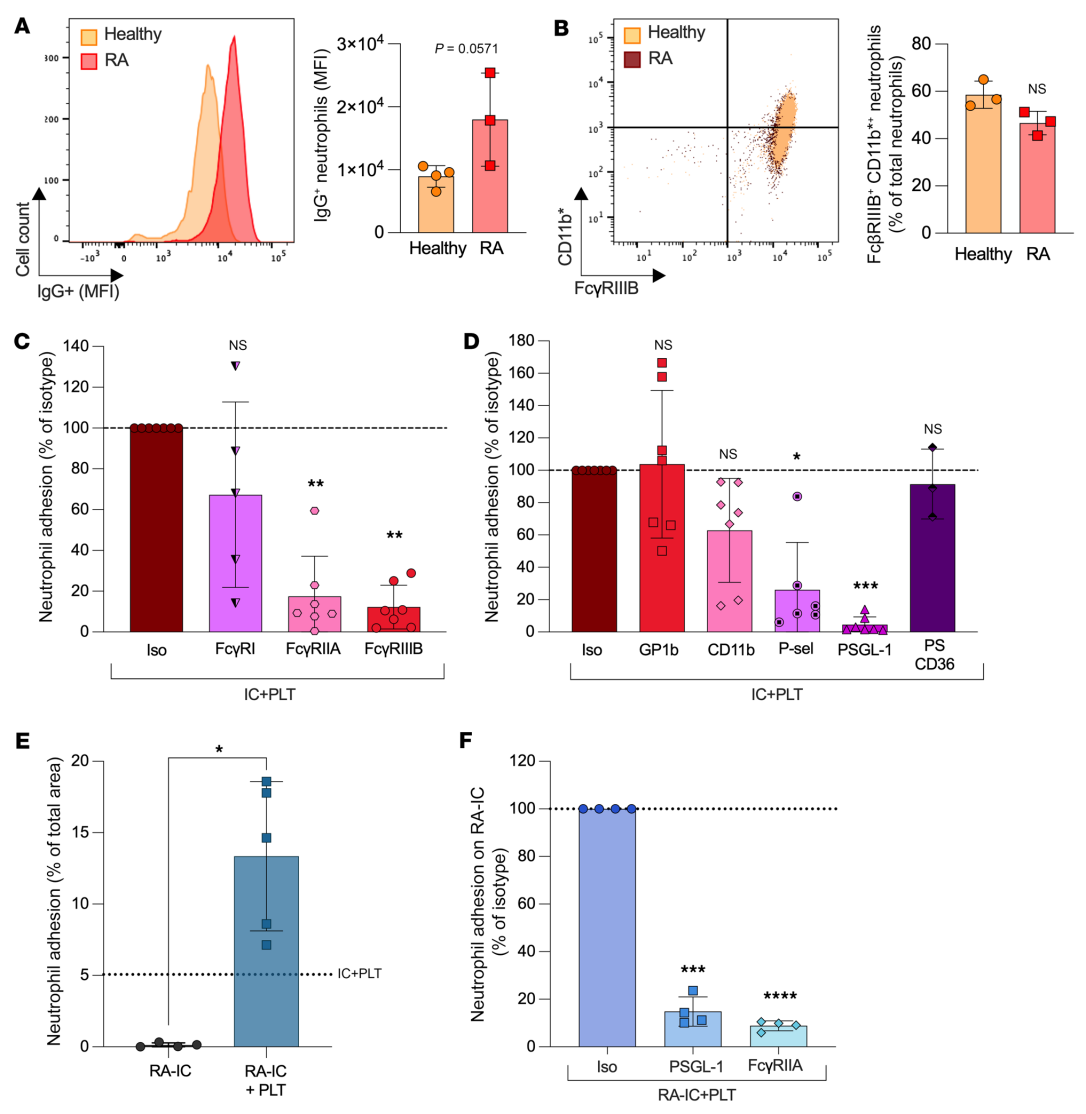
Role of platelets in neutrophil adhesion to ICs in patients with RA. Platelets and neutrophils were isolated from patients with RA and perfused in microcapillaries coated with ICs at 2 dyn/cm^2^. (**A**) IgG-coated neutrophils were evaluated by flow cytometry. Representative histogram overlay of IgG^+^ cells (MFI) from healthy volunteers and patients with RA. Mann-Whitney *U* test (*n* = 3–4). (**B**) Expression of FcγRIIIB and the activated form of CD11b (CD11b*) was measured on neutrophils. A representative dot plot (total overlay) of CD11b* and FcγRIIIB^+^ cells is shown. Mann-Whitney *U* test (*n* = 3–4). (**C**) RA platelets were perfused in IC-coated capillaries and RA neutrophils were perfused in the presence of an isotype or a blocking antibody against Fc receptors. (**D**) Adhered RA platelets were incubated in the presence of an isotype, platelet-blocking antibodies, or a PS blocker, as indicated. RA neutrophils were then perfused in the presence of an isotype or blocking antibodies. The area covered by neutrophils is expressed as the percentage of isotype control. *P < 0.05, **P < 0.01, and ***P < 0.001, by Kruskal-Wallis with Dunn’s test (*n* = 3–7). (**E**) Capillaries coated with ICs from patients with RA (RA-IC) were perfused with or without platelets from healthy volunteers. Neutrophils from healthy volunteers were then perfused. The area covered by neutrophils is expressed as a percentage of the total area. *P < 0.05, by Mann-Whitney *U* test (*n* = 4–5). Mean of neutrophil adhesion on the control (IC+PLT) is indicated with a dotted line. (**F**) Capillaries coated with RA-IC were perfused with or without platelets from healthy volunteers. Neutrophils from healthy volunteers were perfused in the presence of an isotype or blocking antibodies. The area covered by neutrophils is expressed as a percentage of the isotype control. ***P < 0.001 and ****P < 0.0001, by Šídák’s multiple-comparison test (*n* = 4). Data are presented as the mean ± SD.

In individuals with seropositive RA, IgG of various subclasses (predominantly IgG_1_ and IgG_4_) and other antibody isotypes (e.g., IgM, IgA) target the Fc fraction of IgG to form the rheumatoid factor, a type of IC (8, 55). We studied the adhesion of neutrophils on ICs using platelets and neutrophils isolated from individuals recently diagnosed with RA (within 6 months; clinical information is provided in Supplemental Table 1). Neutrophils from these patients already harbored IgG but showed no induced expression of the active form of Mac-1 (Figure 3, A and B, and Supplemental Figure 5). Furthermore, the use of mass cytometry to monitor FcγRIIIB expression by lymphoid and myeloid cellular lineages in blood in these patients compared with healthy controls showed an increase of this receptor in multiple cell types, but not an increase of neutrophils (Supplemental Figure 5). In the microfluidic system, we found that, similar to cells from healthy controls, platelets were crucial in facilitating the binding of neutrophils to ICs. The inhibition of the neutrophil FcγRIIA and FcγRIIIB, but not FcγRI, reduced adhesion by ICs, while inhibition of GPIb or Mac-1 had no effect (Figure 3, C and D). However, the sole blockade of PSGL-1 or P-selectin sufficed to completely inhibit neutrophil adhesion (Figure 3D). Under shear stress conditions, neutrophils bound ICs from patients with seropositive RA more avidly than did those from healthy volunteers; however, this binding still critically required platelets and involved platelet FcγRIIA and PSGL-1 interactions (Figure 3, E and F). Thus, in conditions of autoimmunity, mechanisms of cellular adhesion by ICs heavily depend on bridges composed of PSGL-1 and P-selectin, whereas Mac-1 appears to be dispensable.

**Figure 4:**
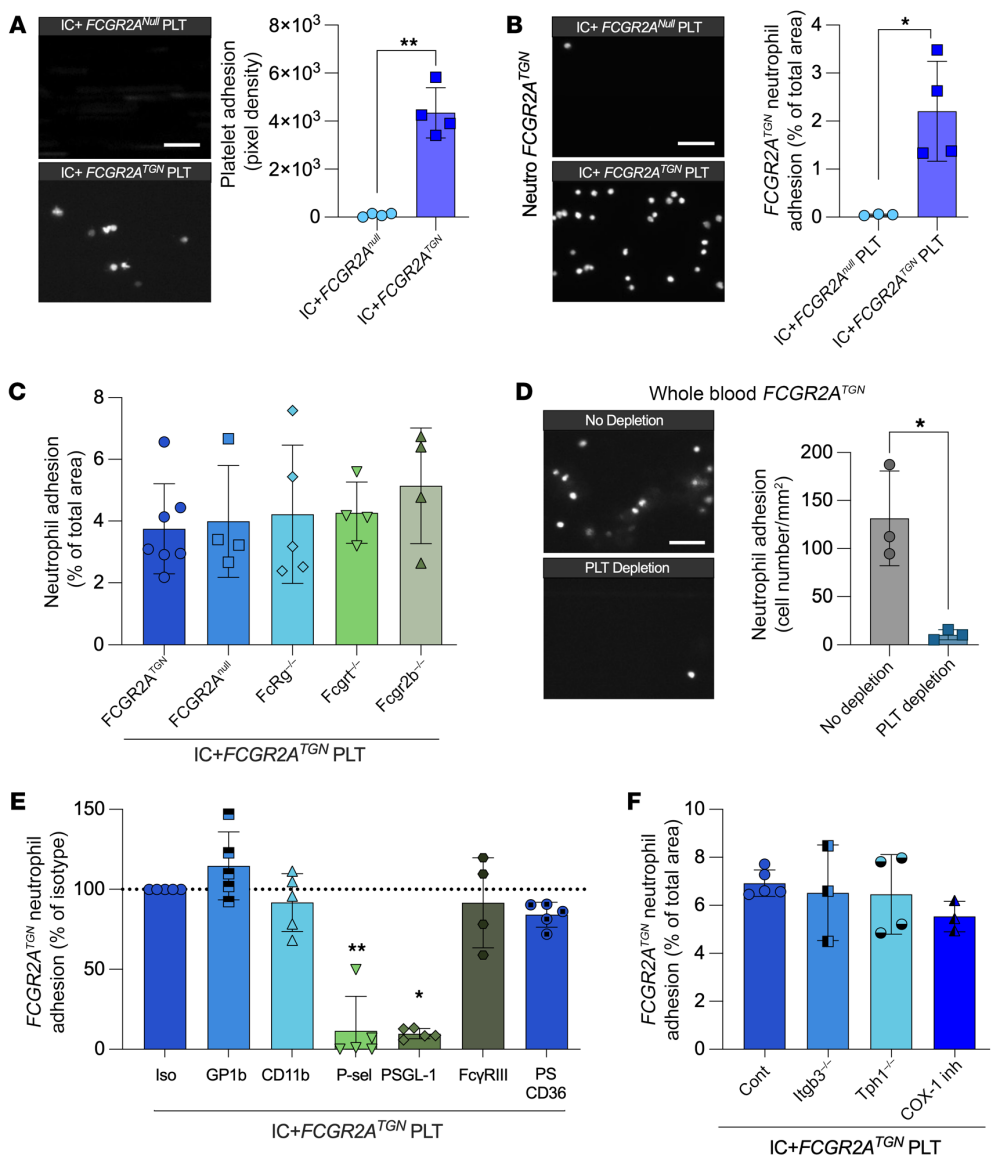
Essential role of mouse platelets in neutrophil adhesion to ICs. (**A** and **B**) Platelets and neutrophils were isolated from mice expressing (*FCGR2A^TGN^*) or not (*FCGR2A^null^*) the FcγRIIA receptor and perfused in microcapillaries coated with mouse ICs at 2 dyn/cm^2^. (**A**) Representative images and quantification of platelet adhesion on ICs (scale bar: 10 μm). **P < 0.01, by unpaired *t* test with Welch’s correction (*n* = 4). (**B**) Representative images and quantification of *FCGR2A^TGN^* neutrophil adhesion on ICs with platelets expressing or not FcγRIIA (scale bar: 40 μm). *P < 0.05, by unpaired *t* test with Welch’s correction (*n* = 3–4). (**C**) *FCGR2A^TGN^* platelets were perfused in mouse IC-coated microcapillaries and neutrophils isolated from different mouse strains were then perfused. *FcRg^–/–^*, γ chain subunit of FcγRI, FcγRIII, and FcγRIV receptor deficiency; *Fcgrt^–/–^*, neonatal Fc receptor deficiency; *Fcgr2b^–/–^*, inhibitory FcγRIIB receptor deficiency. Kruskall-Wallis with Dunn’s test (*n* = 4–5). (**D**) *FCGR2A^TGN^ Ly6gCre^+/–^ Rosa26-TdT^+/–^ Itg2b-YFP^+/–^* mice were depleted or not of platelets (neutrophils expressed dtTomato; platelets expressed YFP), and whole blood (heparin) was perfused in IC-coated capillaries for 10 minutes. Representative images and quantification of neutrophil adhesion on ICs are shown (scale bar: 40 μm). *P < 0.05, by 1-tailed Mann-Whitney *U* test (*n* = 3). (**E**) *FCGR2A^TGN^* platelets were perfused in IC-coated microcapillaries. Adhered platelets were incubated with an isotype, blocking antibodies, or a PS blocker, and *FCGR2A^TGN^* neutrophils were then perfused in the presence of an isotype or blocking antibodies. *P < 0.05 and **P < 0.01, by Kruskal-Wallis with Dunn’s test (*n* = 4–5). (**F**) *FCGR2A^TGN^* platelets deficient for the integrin αIIbβ3 (*Itgb3^–/–^*) or serotonin (*Tph1^–/–^*) or *FCGR2A^TGN^* platelets incubated with COX-1 inhibitor were perfused in IC-coated microcapillaries. *FCGR2A^TGN^* neutrophils were then perfused. Kruskall-Wallis with Dunn’s test (*n* = 3–5). Data are presented as the mean ± SD.

We used these insights to examine the role of these candidate molecules in an in vivo model of antibody-mediated arthritis. In our first set of experiments, we validated the microfluidic assay using mouse cells and ICs. Murine platelets were incapable of binding in these assays because of their complete lack of the FcγR (2). In contrast, mouse platelets isolated from mice expressing the human FcγRIIA gene (*FCGR2A^TGN^*) (56, 57) interacted with immobilized ICs, similarly to platelets from humans (Figure 4A). We therefore utilized platelets from *FCGR2A^TGN^* mice in the following experiments. Neutrophils from *FCGR2A^TGN^* mice isolated from blood by negative selection accumulated on ICs only if *FCGR2A^TGN^* platelets were present (Figure 4B). Intriguingly, the use of mice lacking the Fc common γ chain, thus lacking both FcγRIII and FcγRIV, as well as mice lacking the neonatal FcR (FcRn) or the inhibitory FcγRIIB, along with comparisons of neutrophils isolated from *FCGR2A^null^* and *FCGR2A^TGN^* mice, revealed that these FcγRs on the neutrophils’ side were dispensable when platelets were present. This was not attributed to specific characteristics that neutrophils might have gained through maturation in the blood, as the utilization of neutrophils isolated from the bone marrow validated similar results (Figure 4C and Supplemental Figure 6). Furthermore, we used blood from *FCGR2A^TGN^ Ly6g^Cre+/–^ Rosa26-*TdT*^+/–^* *Itga2b-*YFP*^+/–^* quadruple-transgenic mice, in which neutrophils and platelets fluoresce red and yellow, respectively (58). Using whole blood from these isotype-treated or platelet-depleted reporter mice, we further validated that platelets are strictly required for neutrophil binding to ICs and that this function cannot be substituted by any other circulating cells or blood component (Figure 4D).

The reliance on FcγRIIA-expressing platelets to recruit neutrophils prompted our investigation of platelet-derived molecules supporting platelet interactions with neutrophils in mice. To improve neutrophil recovery, cells were isolated from platelet-depleted mice, as platelet-bound neutrophils were frequently observed in controls and reduced the yield (mean ± SD: 0.51 ± 0.28 × 10^6^/mL in depleted vs. 0.19 ± 0.06 × 10^6^/mL in controls; *n* = 5). Importantly, platelet depletion did not affect downstream analyses (Supplemental Figure 7). As in patients with RA, the blockade of PSGL-1 completely inhibited neutrophil adhesion on ICs, while antibodies targeting GPIb or Mac-1 had no effect (Figure 4E). Activated αIIbβ3, the platelet integrin that binds fibrinogen, has been shown to amplify FcγRIIA intracellular signaling and degranulation following activation by ICs (4, 45, 59). By using platelets from *FCGR2A^TGN^ Itgb3^–/–^* mice, we found that αIIbβ3 was dispensable in neutrophil adhesion by platelets (Figure 4F), thus ruling out its role in signaling as well as in bridging platelets and neutrophils through fibrinogen, Mac-1, or SLC442A (38, 41).

FcγRIIA signaling relies on the immunoreceptor tyrosine-based activation motif (ITAM) family of receptors (60). Among all receptors studied in platelets that contain the immunoreceptor tyrosine–based inhibition motif (ITIM), the receptors G6b-B and TREM-like transcript 1 (TLT-1) demonstrate the most important regulatory activity in vivo (61–63). Because FcγRIIA is not found in mice, the role of the ITIM receptors in regulating FcγRIIA activation has never been examined to our knowledge. We thus crossed *FCGR2A^TGN^* mice with *Mpig6b*^–/–^ or *Treml1*^–/–^ mice, lacking G6b-B and TLT-1 respectively, and examined the efficiency of *FCGR2A^TGN^* *Mpig6b*^–/–^ and *FCGR2A^TGN^* *Treml1*^–/–^ platelets to immobilize on ICs and recruit neutrophils. While the absence of G6b-B, but not of TLT-1, enhanced platelet binding to ICs (Supplemental Figure 8), we found that both these ITIM receptors were dispensable in the IC-mediated adhesion of neutrophils by platelets (Supplemental Figure 8). Serotonin, produced from the tryptophan hydrolase 1 (Tph1) enzyme in the intestine and captured abundantly in platelet-dense granules (64), supports neutrophil migration to inflamed sites in mice (65). Moreover, thromboxane A_2_, which requires the activity of cyclooxygenase 1 (COX-1) for its biosynthesis (66, 67), amplifies platelet activation. With the use of *FCGR2A^TGN^* *Tph-1^–/–^* and COX-1 inhibition by SC-560, we found that secretion of serotonin and metabolism of arachidonic acid into prostaglandins were dispensable in both platelet binding to ICs and neutrophil adhesion (Figure 4F and Supplemental Figure 9). Thus, in mice, FcγRIIA-expressing platelets support neutrophil adhesion on immobilized ICs in a process that critically relies on neutrophil PSGL-1. These findings are reminiscent of those observed in patients with RA.

**Figure 5:**
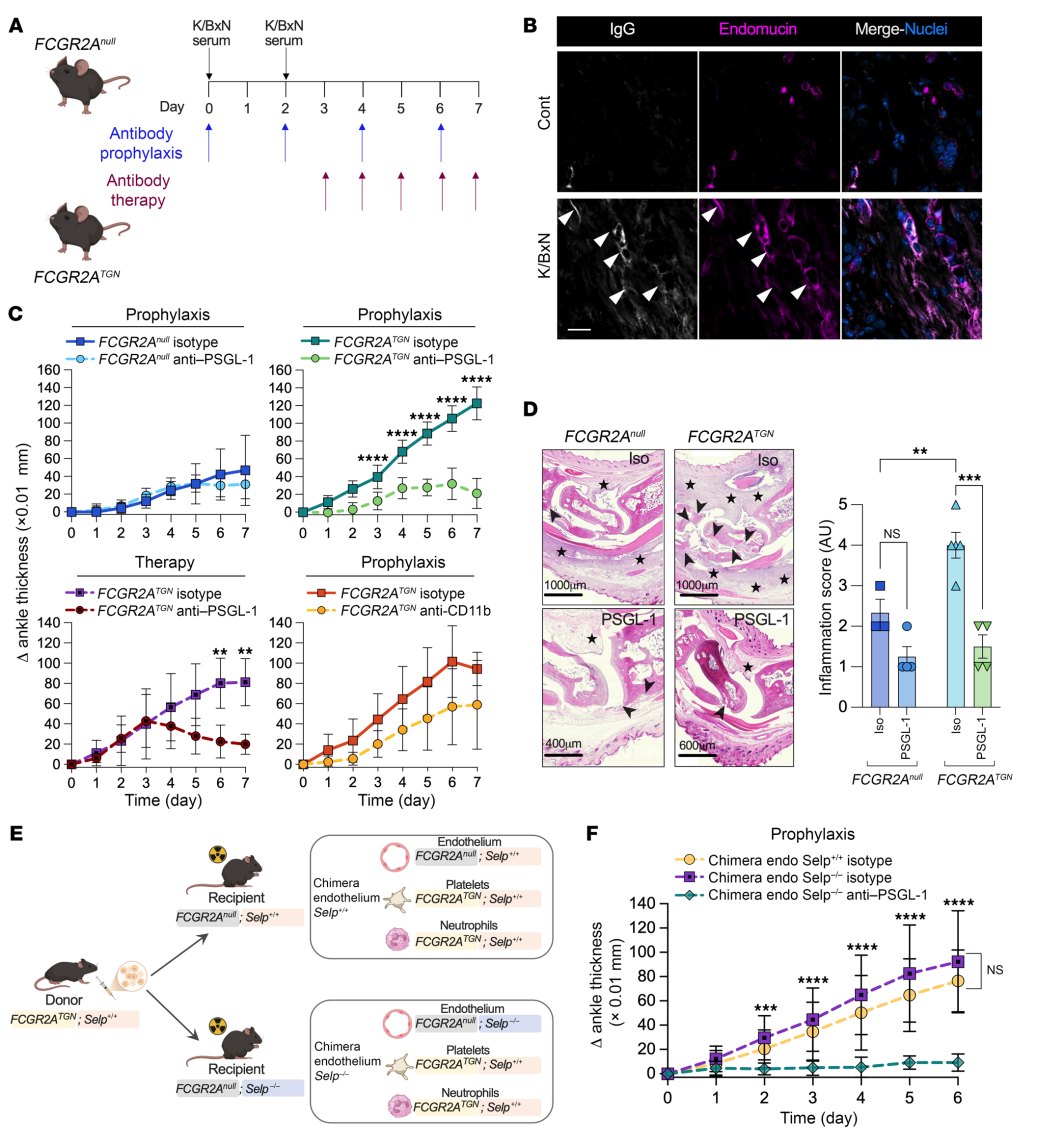
Role of the combination of FcγRIIA and PSGL-1 in autoantibody-mediated arthritis pathogenesis. (**A**) Representation of the K/BxN arthritis model induced with two i.p. injections of K/BxN serum. An isotype or blocking antibody was injected i.p., every other day beginning on day 0 (D0) for prophylactic treatment or everyday, beginning on D3 for therapeutic treatment. (**B**) IgG depositions were evaluated by immunofluorescence in the joint vasculature (endomucin) of *FCGR2A^TGN^* mice after 7 days of arthritis (K/BxN) or in nonarthritic mice (CONT). White arrowheads indicate IgG deposition in capillaries. Images are representative of 3 mice (scale bar: 15 μm). (**C**) Disease severity was monitored daily by measurement of ankle thickness. Blocking antibodies were injected into *FCGR2A^null^* and *FCGR2A^TGN^* mice. **P < 0.01 and ****P < 0.0001, by multiple Mann-Whitney *U* tests. (*n* = 7–10 per group). (**D**) Arthritic joints from *FCGR2A^null^* or *FCGR2A^TGN^* mice were analyzed. Antibodies were injected as a prophylactic treatment. The inflammation score was measured after H&E coloration. Cell infiltration (star) and cartilage loss (arrowheads) are indicated. A representative image of each group is shown (scale bars: ) **P < 0.01 and ***P < 0.001, by 1-way ANOVA with comparisons (*n* = 3–5). (**E**) Experimental design to obtain chimera mice with or without P-selectin (*Selpl*) expression on the endothelium and expressing both FcγRIIA and P-selectin on myeloid cells. (**F**) Ankle thickness was evaluated in chimera mice with or without *Selp1* expression on the endothelium. Isotype or blocking antibodies were injected into P-selectin–deficient chimera mice expressing both FcγRIIA and P-selectin on myeloid cells. The isotype was injected into *FCGR2A^null^* chimera mice expressing both FcγRIIA and *Selp1* on myeloid cells. Antibodies were administrated as a prophylactic treatment in arthritic mice. ***P < 0.001 and ****P < 0.0001, by 2-way ANOVA with Tukey’s test (*n* = 5–8 per group). Data are presented as the mean ± SD.

To determine the significance of these findings in IC-mediated pathogenesis, we utilized the K/BxN serum transfer model of arthritis (Figure 5A) (68). This model involves the formation of ICs that are composed of IgG-targeting circulating glucose-6-phosphate isomerase and that deposit in the joints (8, 69), with contributions of both neutrophils and platelets (70, 71). Microscopic analyses of the harvested arthritic joints confirmed IC depositions in small vessels (estimated mean diameter of 12.1 μm ± 5.4 [SD]) (Figure 5B). Arthritis was induced in *FCGR2A^null^* and *FCGR2A^TGN^* mice, and disease progression was followed over time. Moreover, to establish the role of PSGL-1 in arthritis pathogenesis, either anti–PSGL-1–blocking antibody or an isotype antibody was injected into these mice. Disease severity was aggravated in *FCGR2A^TGN^* mice, whereas PSGL-1 blockade nearly completely abrogated the pathogenesis (Figure 5C). In contrast, inflammation in *FCGR2A^null^* mice was modest and was not significantly affected by PSGL-1 blockade (Figure 5C). The blockade of PSGL-1 has therapeutic potential, as it reduced inflammation scores and ankle thickness of *FCGR2A^TGN^* mice when injected into mice with established arthritis (Figure 5, C and D).

Although P-selectin is principally expressed by platelets, it can be exposed by endothelial cells in inflammatory conditions (72). To dissect the role of endothelial cell–derived P-selectin, while preserving FcγRIIA expression in platelets, we generated bone marrow chimeric mice (Figure 5E). P-selectin–deficient (*Selp^–/–^*) and WT (*Selp^+/+^*) mice were lethally irradiated and reconstituted with bone marrow from *FCGR2A^TGN^* donors. In this setup, platelets express both FcγRIIA and P-selectin, whereas endothelial cells in *Selp^–/–^* recipients lack P-selectin. Both *Selp^–/–^* and *Selp^+/+^* chimeras developed arthritis to a similar extent (Figure 5F). Notably, PSGL-1 blockade completely abolished arthritis in these mice (Figure 5F), indicating that endothelial P-selectin is dispensable and that PSGL-1 inhibition thus most likely disrupts platelet-neutrophil interactions. In contrast, the blockade of Mac-1 failed to reduce the course of arthritis (Figure 5C), confirming that the key molecules involved in platelet and neutrophil interactions were also involved in the promotion of inflammation in vivo.

**Figure 6:**
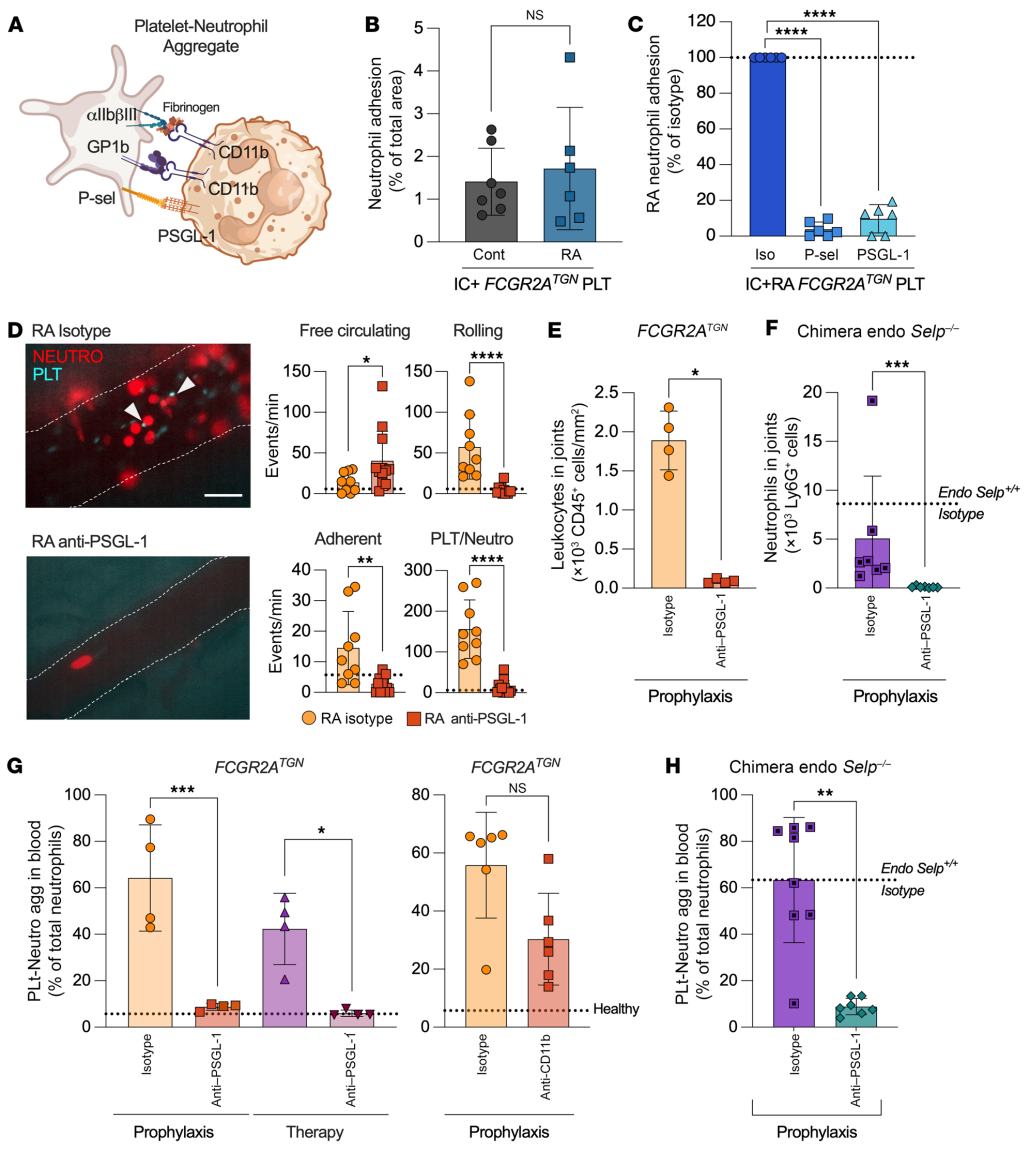
Platelets and neutrophils associate through PSGL-1 in inflammatory arthritis. (**A**) Platelet-neutrophil interactions targeted by blocking antibodies and their ligands are shown. (**B** and **C**) Cells were isolated from arthritic (RA) or nonarthritic (Cont) *FCGR2A^TGN^* mice and perfused in IC-coated microcapillaries. (**B**) Neutrophil adhesion in RA and control conditions. Mann-Whitney *U* test (*n* = 6–7). (**C**) Adhered RA platelets were incubated with an isotype or blocking antibody followed by RA neutrophil perfusion in the presence of isotype or blocking antibodies. ****P < 0.0001, by 1-way ANOVA with Dunnett’s test (*n* = 6–7). (**D**) Arthritis was induced for 3 daysin *FCGR2A^TGN^ Ly6gCre^+/–^ Rosa26-TdT^+/–^ Itg2b-YFP^+/–^* mice injected or not with an anti–PSGL-1–blocking antibody. Intravital imaging of joints was performed. Representative images show neutrophil-platelet interactions (arrowheads) (scale bar: 15 μm). Healthy levels are indicated with dotted lines (*n* = 2). *P < 0.05, **P < 0.01, and ****P < 0.0001, by Mann-Whitney *U* test (*n* = 3–4). (**E** and **F**) Leukocyte infiltration (**E**) into arthritic joints was evaluated by microscopy and neutrophil infiltration (**F**) by flow cytometry in *Selp*-deficient chimera mice expressing both FcγRIIA and P-selectin on myeloid cells. *P < 0.05 and ***P < 0.001, by Mann-Whitney *U* test (*n* = 4–7). (**G**) Blocking antibodies were injected into *FCGR2A^TGN^* arthritic mice as indicated (prophylaxis or therapy), and platelet-neutrophil aggregates (PLT-Neutro agg) were evaluated. The level of aggregates was measured in healthy mice and is indicated with a dotted line. *P < 0.05 and ***P < 0.001, by 1-way ANOVA with Tukey’s test (*n* = 4, left) and Kolmogorov-Smirnov test (*n* = 6, right). (**H**) Platelet-neutrophil aggregates were measured in blood from arthritic *Selp*-deficient chimera mice expressing both FcγRIIA and P-selectin on myeloid cells and injected with isotype or anti–PSGL-1–blocking antibody as a prophylactic treatment. **P < 0.01, by Mann-Whitney *U* test (*n* = 7–8). Data are presented as the mean ± SD.

To study cellular interactions in these arthritic conditions, platelets and neutrophils were isolated from the blood on day 7, i.e., at the peak of inflammation, for our ex vivo microfluidic investigations (Figure 6, A–C). In our microfluidic system, we confirmed that in murine arthritis, as in patients with arthritis, neutrophils critically depended on interactions with platelets through P-selectin and PSGL-1 bridges (Figure 6, B and C).

We investigated the consequences of PSGL-1 inhibition using intravital imaging in *FcγRIIA^TGN^ Ly6g^Cre+/–^ Rosa26-TdT^+/–^ Itg2b-YFP^+/–^*–transgenic mice (58). We observed frequent neutrophil rolling, extended neutrophil interactions with the blood vessels, and platelet-neutrophil interactions in the arthritic joints of arthritic mice, but almost never in the control animals (Figure 6D and Supplemental Videos 3 and 4). Importantly, these interactions, visualized at day 3 following the injection of arthrogenic serum, were completely absent in mice injected with a anti–PSGL-1–blocking antibody (Figure 6D and Supplemental Video 5). Moreover, the quantification of neutrophils in arthritic joints confirmed the critical role of FcγRIIA and of the interaction of platelet-derived P-selectin with PSGL-1 in the recruitment of neutrophils to the inflamed tissue (Figure 6, E and F).

In RA as well as in numerous inflammatory conditions, platelet-neutrophil aggregates are detected in the blood (43). We thus monitored platelet-neutrophil aggregates in the blood of these mice (Figure 6G) and found that platelet-neutrophil interactions were more abundant in arthritic *FCGR2A^TGN^* mice than in healthy mice. These interactions were completely abrogated by the blockade of PSGL-1 interaction with platelet-derived P-selectin (Figure 6, G and H). In contrast, the blockade of Mac-1, which had no effect in our microfluidic analyses, failed to significantly reduce platelet-neutrophil aggregates or the course of arthritis (Figure 6G). Together, these findings suggest that platelet-neutrophil aggregates in the blood indeed mirror such cell interactions taking place locally in arthritic joints. Furthermore, our observations confirm that FcγRIIA-expressing platelets interacted with neutrophils in a process that required platelet-derived P-selectin and PSGL-1, but not Mac-1, in arthritis in vivo.

## Discussion

Neutrophils are the most abundant leukocytes in the blood circulation. Cues from the circulation, such as granulocyte-macrophage colony-stimulating factor (GM-CSF) and osteopontin, or platelet-derived molecules such as platelet factor 4 and RANTES, can dictate neutrophil production and egress from the bone marrow (73, 74). They are short lived and do not reside in tissues; therefore, there must be signals that instruct neutrophils on how to exit the vasculature to invade inflamed tissues. In flow conditions, activation of endothelial cells, as well as IC deposition have been demonstrated to be key mechanisms promoting neutrophil rolling and adhesion, which are necessary steps in the initiation of diapedesis (19, 75, 76). Our findings identify what we believe to be a previously unrecognized model by which neutrophils can resist shear stress that critically requires platelets in a context involving autoantibody deposition. Traditionally viewed as bystanders that become activated secondary to inflammation or tissue damage (e.g., collagen exposure or cytokines), platelets were shown here to act as early responders in autoimmunity. Disrupting this interaction prevented neutrophil-platelet engagement and effectively blocked inflammation in a model of autoimmune arthritis.

Hydrodynamics in the bloodstream forces neutrophils and other larger leukocytes to circulate principally in the center of the lumen, with limited access to the vessel wall (77, 78). However, the abundance of platelets in the bloodstream, coupled with their intricate structure (flat disc, absence of a nucleus) and arrays of molecules facilitating both adhesion to the vasculature as well as interactions among platelets, render them exceptionally well suited to survey and adhere to the vessel wall within the marginal flow region where there are more demanding shear stress conditions (31). Intriguingly, the use of spherocytic platelets to evaluate the importance of the platelet discoid shape revealed that this shape was unnecessary to adhere to the vascular wall in a FeCl_3_ model or microchambers using immobilized collagen (79). In contrast, engineered discoid platelet-like nanoparticles exhibit enhanced adhesion compared with their spherical counterparts (80). The platelet discoid shape appeared more recently in the course of evolution in mammals (81, 82). However, whether this feature plays a role in immunity through platelet adhesion to ICs is unknown. In arterial blood, platelets may adhere strongly to exposed extracellular matrix or vWF when blood vessels are damaged and can even form a thrombus despite shear stress as high as 70 dyn/cm^2^ (83, 84). Such interactions involve abundantly expressed receptors (e.g., GPIb-V-IX, 25,000 copies; αIIbβ3, up to 80,000 copies) (85, 86) and more likely resist against much higher shear stress than what we could measure when ICs were utilized as immobilized ligand. The relatively low resistance to shear stress in our study may have been due to the lower density of FcγRIIAs (400–2,000 copies) (87) or to potential shedding of this receptor upon platelet activation (88).

Monomeric IgG failed to mediate platelet adhesion, possibly because FcγRIIA is a low-affinity receptor (2, 89) that allows constant association and dissociation with IgG, resulting in a preference for binding hexameric IgG structures (90). Such association and dissociation may protect platelets from continuous activation, as they constantly bath in an antibody-rich environment. We showed that platelets resisted shear stresses up to 2 dyn/cm^2^ (Figure 1). This is consistent with the forces encountered in microvessels, such as those in joints or organs, and in atherosclerosis-prone arterial regions (91). In autoimmune diseases, additional mechanisms may also recruit platelets, enabling them to support neutrophil recruitment. This could explain how antibody-mediated diseases have been studied in mice despite the biological bias that murine platelets lack the FcγRIIA. Beyond direct binding to deposited ICs, activated endothelial cells, whether triggered by endotoxins or adjuvants commonly used in mouse models, or inflammation itself, may expose selectins or vWF, both of which promote platelet recruitment (92, 93). Neutrophils and platelets may recognize ICs through direct binding to antigens, such as citrullinated proteins found in RA, for example (8). Damages to the vasculature may also expose extracellular matrix components, further facilitating platelet adhesion. Consistent with this, platelet glycoprotein VI, an ITAM receptor like FcγRIIA and a collagen receptor (60), enhanced joint inflammation in our arthritis model (71) and was found contributing to neutrophil recruitment and NETosis in acute lung injury (94). Although neutrophil extracellular traps (NETs) were increased in blood circulation in our mouse model, they were not significantly reduced by PSGL-1 inhibition and were not significantly elevated in the in vitro system (Supplemental Figure 10). It may thus be relevant to decipher the distinct mechanisms that may promote NETosis in inflammatory contexts.

Studies have demonstrated that mechanisms involving endothelial cell activation and recognition of IC deposits allow neutrophils to roll and adhere on the vascular wall under conditions of shear stress. Interactions with the vessel wall can involve selectins and integrins such as Mac-1 binding to intercellular adhesion molecule 1 (ICAM-1) (19), and may be favored in narrower vessels, such as those in the microcapillaries that supply the lungs (95). The role of IC deposits has been elegantly characterized with human cells in a microfluidic system. It involves neutrophil tethering by FcγRIIIB, which would allow initial neutrophil interactions with IgG, followed by the formation of more solid bonds between FcγRIIA and IgG (21, 26–29). Our findings confirm this established model, while integrating a role for platelets and defining the overlooked molecular mechanisms promoting platelet-neutrophil interactions. As for the blockade of FcγRI, the strong interpatient variability suggests that this receptor may play a role in IC interactions in some patients with RA. This would be consistent with the reported intracellular localization of FcγRI and its surface exposure following neutrophil activation by ICs (96).

We propose that FcγRIIIB and FcγRIIA may require Mac-1 activation, which may play a role independent of its binding to platelet GPIb (49, 97). However, neutrophil interactions with ICs may not suffice to promote arrest and optimal adhesion to ICs and may require P-selectin from the immobilized platelets. This was supported by our observation of multiple waves of calcium mobilization in neutrophils injected into the microfluidic system, which points to immobilized ICs inducing sequential activation of neutrophils (Supplemental Figure 11 and Supplemental Video 6). This would result in neutrophils attaching to ICs in the vicinity of platelets, thus explaining why the large majority of the neutrophils that we visualized colocalized with platelets (Figure 1E). In RA, circulating neutrophils are already exposed to IgG and cytokines, suggesting that activation might have already occurred in the blood and that a different mechanism promotes recruitment by immobilized ICs. Although we failed to detect significant amounts of the activated form of Mac-1, this integrin was mostly dispensable on cells from patients with RA, suggesting that FcγRIIIB and FcγRIIA can interact with ICs due to other activation processes in RA. In mice, the role for platelets has so far been completely overlooked, as mouse platelets do not express any FcγRs and thus cannot interact with ICs (2). By using cells from patients with RA and transgenic mouse cells to more adequately mimic human pathogenesis, we identified the essential role of platelets in IC-mediated pathogeneses.

ICs may not only form in tissues, but may also accumulate in blood, since administration of drugs or vaccines may also induce IC generation, thereby promoting thrombosis and reduced platelet counts. Notably, heparin and certain SARS-CoV-2 vaccines can stimulate the formation of platelet factor 4–containing (PF4-containing) ICs (98–105). Platelets can also recruit neutrophils into thrombi, aggravating thromboinflammation. This process can also implicate IgG, but in a FcγR-dependent manner, in addition to IgM and complement activation (92). Such neutrophil-rich thrombi can occlude pulmonary arteries, veins, and the microvasculature (106). In gut ischemia, damage to multiple organs can result from the neutrophils’ CD36 binding to platelet PS. The neutrophils then rip away large PS-rich membrane fragments, thus favoring neutrophil microaggregates (42). Although we ruled out a role of CD36 in our model, we did not assess the contribution of other PS-binding molecules, such as Gas6 or MFG-E8 (107, 108). PS was exposed on platelets in our model but proved dispensable in neutrophil-platelet interactions (Figures 2 and 3), which might be consistent with the fact that RA is not a thrombotic disorder per se. PS exposure, however, is a prerequisite for extracellular vesicle release by platelets. In response to ICs in the microfluidic system, platelets, but not neutrophils, released extracellular vesicles, the majority of which exposed both P-selectin and PS. Even in the absence of platelets, these vesicles enriched by centrifugation were capable of supporting neutrophil recruitment by ICs (Supplemental Figure 12). Although platelet-derived extracellular vesicles were not found to be increased in blood in our murine arthritis model (Supplemental Figure 12), they may be produced locally in the joint vasculature, which would be consistent with their accumulation in the synovial space in humans and mice with arthritis through a more permeable vasculature (71, 109). These findings further support the necessary role of P-selectin and suggest that the role of extracellular vesicles may go beyond the promotion of inflammation, given their cargo rich in molecules such as damage-associated molecular patterns or inflammatory IL-1 (46, 71, 110).

In the present study, we did not evaluate the role of platelet receptors, such as CD40L or TREM-1 ligand, that may support neutrophil interactions (39, 40, 111, 112). Moreover, there may be other such molecules that have not yet been identified. Nevertheless, we ruled out the involvement of several candidate molecules — namely αIIbβ3, GPIb, and Mac-1 (and, by extension, SLC44A2 and JAM-3) — in mediating platelet binding to ICs and neutrophil recruitment. The only exceptions were P-selectin and its ligand PSGL-1, both of which proved essential. Stimulation of G6b-B inhibits platelet function by inducing downstream signaling through tyrosine phosphatases (62, 113). Mice that lack TLT-1 develop elevated levels of D-dimers and cytokine TNF in plasma following the injection of LPS (63). It had previously never been verified whether these ITIM-containing receptors could modulate FcγRIIA. By using *FCGR2A^TGN^ Mpig6b^–/–^* and *FCGR2A^TGN^ Treml1^–/–^* platelets, we found that they were dispensable, which suggests that they might regulate other platelet functions unrelated to IgG interactions. In line with the observations made in the microfluidic system, the *FCGR2A^TGN^* *Treml1^–/–^* mice developed arthritis similarly to those that had maintained TLT-1 expression (Supplemental Figure 13). Note that although we revealed that the absence of G6b-B enhanced platelet accumulation on ICs, which points to a role in the regulation of FcγRIIA, *Mpig6b*^–/–^ mice are macro-thrombocytopenic (62, 114), which prevented us from confidently determining whether G6b-B is involved in arthritis in vivo.

While serotonin and thromboxane A_2_ were also found to be dispensable in neutrophil adhesion to ICs, they may play other roles in the pathogenesis, such as by stimulating neutrophil diapedesis or loosening endothelial junctions (115). Serotonin promotes neutrophil transmigration (65, 116) and can promote the joint vasculature leakage in arthritis (59, 109). Arachidonic acid, either produced by platelets or transferred from activated neutrophils (117), can be metabolized into thromboxane A_2_ by cyclooxygenase-1 in platelets. Inhibition of cyclooxygenase-1 in platelets had no effect on neutrophils; however, research has indicated that fibroblast-like synoviocytes, which participate significantly in arthritis, metabolize PGH_2_, an intermediate of thromboxane A2 into pathogenic prostacyclin through an intercellular mechanism (118).

There are limitations to our studies. While the microfluidic approach allowed us to examine intricate mechanisms involving Fc receptor binding human cells, including those from patients with RA, it focused on platelets and neutrophils and involved aggregated IgG. The use of whole blood validated our conclusions, but it excluded potential contributions of endothelial cells. The use of an arthritic mouse model that strictly relies on the formation of antigen-specific immune complexes, together with intravital imaging and an irradiation chimera approach, however, eliminated the contribution of endothelial cell–derived P-selectin in the arthritis process. Furthermore, platelets were similarly necessary when monocytes, instead of neutrophils, were included in the in vitro assays (Supplemental Figure 14), pointing to a conserved mechanism among leukocytes. Moreover, there are also significant differences between humans and mice in terms of recognition of ICs by FcγRs. Unexpectedly, all neutrophil FcγRs tested were dispensable in mice once FcγRIIA-expressing platelets were present, which is further evidence of interspecies differences. Whether these receptors contribute to redundancy, or whether there is a role for IgM receptors through binding of hexameric IgG complexes (92) has not been verified to our knowledge. However, the use of *FCGR2A^TGN^* mice and samples (cells and antibodies) from patients with RA has allowed us to confirm the role of the key molecules we had identified in humans, i.e., FcγRIIA and PSGL-1, in a mouse model of arthritis.

Neutrophils can also recruit platelets to inflammatory sites (119), such as in cremaster muscles of mice treated with the cytokine TNF, a process also requiring PSGL-1 (34). Neutrophils recruit platelets in the reversed Arthus reaction in the skin, which involves ITAM signaling in platelets (120, 121). These models did not include antibody binding to FcγRIIA, because the latter receptor was not expressed in these mice; however, they point to the conserved nature of platelet-neutrophil–orchestrated activities in multiple mechanisms of inflammation. But what is the purpose of platelet recruitment of neutrophils onto IC depositions, since the aggravation of autoimmune diseases is unlikely to reflect the evolutionary function of the platelets’ response? Perhaps neutrophils, and other FcγR-expressing cells, are needed to combat microbial agents opsonized by IgG, and platelets help with this process. Moreover, we cannot exclude the possibility that FcγRIIA also contributes to platelet adhesion to a damaged vasculature in the absence of autoimmunity, similar to the way that natural antibodies from innate B cells frequently recognize molecules such as collagen, fibrinogen, or nuclear proteins that might be deposited in a wound (122). A similar process may thus recruit neutrophils to the site of injury through humoral immunity.

Certain *FCGR2A* gene haplotypes are associated with increased risks of autoimmune diseases (89). Moreover, the addition of the *FCGR2A* transgene in mice aggravates sepsis, anaphylaxis, and lupus nephritis (4, 123, 124). It is tempting to speculate that blocking neutrophil recruitment by platelets could be advantageous in multiple pathogeneses involving ICs. Our research has thus identified an underappreciated mechanism by which platelets support neutrophil functions, with significant relevance to autoimmunity.

## Methods

### Sex as a biological variable

Patients with RA were recruited with a female-to-male ratio of approximately 66:33. Mass cytometry analyses were matched for sex and age with healthy controls, and all microfluidic experiments were sex matched. No formal analyses were performed to assess sex as a biological variable due to limited statistical power. For in vivo experiments, only male mice were used, as they consistently developed more robust and reproducible arthritis in this model.

Additional details on methods can be found in the Supplemental Methods.

### Microfluidic experiments

Microfluidic experiments were performed with a BioFlux 1000HT system (Cell Microsystems) coupled to a Zeiss Axio Observer Z1 microscope and a Hamamatsu CMOS camera acquisition system. The BioFlux 1000HT system consists of a single pump controlling a constant fluid flow rate of 0.5 to 200 dyn/cm^2^ adjusted to the viscosity of the perfused liquid to obtain experimental conditions close to the physiological context found in rodents and humans. Channels of a 24-well BioFlux plate (Cell Microsystems, 910-0047) were coated overnight at room temperature (RT) with either 500 μg/mL human IC (MP Biomedicals, 0855908), 500 μg/mL mouse IC (MilliporeSigma, I5381), 1% BSA (negative control), 500 μg/mL human monomeric IgG, or 100 μg/mL fibrinogen (Enzyme Research Laboratories, FIB-3). Capillaries were saturated with 1% BSA for 2 hours at RT and washed using Tyrode’s Buffer, pH7.4 (TB7.4). ICs were obtained by heat aggregation of monomeric IgG (25 mg/mL) at 62°C for 1 hour and ICs from patients with arthritis (RA-ICs) were obtained by heat aggregation of immunoglobulins (5 mg/mL) isolated from pooled sera from 3 rheumatoid factor–seropositive patients and purified on sepharose 4B. Platelets labeled with CellTracker Orange (CMRA, Invitrogen, Thermo Fisher Scientific, C34551) or control solution (TB7.4) were flowed for 30 minutes in the presence of 5 mM CaCl_2_ at different shear stresses (0.5, 1, 2, or 3 dyn/cm^2^). In some experiments, platelet extracellular vesicles (150 μg/mL) were added instead of platelets. Capillaries were washed using TB7.4 containing 5 mM CaCl_2_ in the presence or absence of platelet-blocking antibodies (Supplemental Table 2) or a COX-1 inhibitor (SC-560, 100 nM, Cayman Chemicals, 70340) for 10 minutes. Neutrophils labeled with 2 μM CellTracker Green (CMFDA, Invitrogen, Thermo Fisher Scientific, C7025) or FLUO-4 (1 μM, 30 minutes at RT, Invitrogen, Thermo Fisher Scientific, F14217) were flowed with 5 mM CaCl_2_ in the presence or absence of blocking antibodies at different shear stresses (0.5, 1, 2, or 3 dyn/cm^2^). For quantification, ImageJ software (NIH) was used to draw a region of interest (ROI) corresponding to the capillary. A threshold was applied so that only pixels falling within the platelet or neutrophil range were found. The software converts all pixels under the threshold to black and all pixels above the threshold to white. The platelet pixel area above threshold (in square pixels) was measured and defined as the pixel density. The neutrophil pixel area was measured and expressed as a percentage of the total area covered by neutrophils. Platelet adhesion at 30 minutes was quantified by CMRA fluorescence acquisition at ×400 magnification (dsRed settings), and neutrophil adhesion was quantified by CMFDA fluorescence acquisition at ×100 magnification (FITC settings) every 30 seconds for 15 minutes.

### Statistics

Data are presented as the mean ± SD. Datasets were compared for statistical significance using GraphPad Prism 10 software, and a *P* value of less than 0.05 was considered significant. Student’s *t* test and Mann-Whitney *U* test were both performed as 2-tailed tests, unless otherwise indicated in the figure legend. ANOVA was used for 1 independent variable and at least 3 groups and 2-way ANOVA for 2 independent variables. For all in vivo experiments, histological quantifications and Bioflux quantifications, the analyses were performed in a blinded manner.

### Study approval

#### Humans.

The Clinical Research Ethics Committee of the CHU de Québec – University Laval approved our study (CHU de Québec – Université Laval REB no. B14-08-2108). Blood was obtained from healthy adult volunteers recruited through the clinical research facility at the Centre de Recherche CHU de Québec – University Laval. Patients with RA were recruited through the CHU de Québec – Université Laval Systemic Autoimmune Rheumatic Diseases Biobank and Repository Database. The CHU de Québec – Université Laval Ethics Committee has approved the biobank and its management (CHU de Québec-Université Laval REB no. B13-06-1243). Demographic and clinical factors for patients with arthritis are summarized in Supplemental Table 1. Healthy volunteers and patients with RA provided written, signed consent forms prior to blood collection.

#### Mice.

This study followed the guidelines of the Canadian Council on Animal Care. The protocol was approved by the Animal Welfare Committee at Laval University (CHU 2023-1290).

### Data availability

Data associated with this study are present in this manuscript or the supplemental materials. The values for all data points shown in graphs or behind reported means are available in the Supporting Data Values file.

## Author contributions

Experiments were conceived and designed by MB, IA, ED, MV, and EB. TL, NV, NB, VAW, YS, PD, SL, PF, and CB contributed critical reagents, resources, and expertise. Experiments were performed by MB, IA, ED, MV, and TL. Data were processed and analyzed by MB, IA, ED, MV, MM and supervised by EB. The manuscript was written by MB, IA, and EB and critically reviewed by all authors. There are 2 co–first authors, and MB was determined as first co-author, given her trainee status at the time of performing the experiments.

## Funding support

The Arthritis Society (TAS) and the Canadian Institutes of Health Research (CIHR) grants (to EB, CB, PF, and SL).

- Fonds de Recherche en Santé du Quebec Merit Award (FRQS) (to EB).
- FRQS Senior Award (to CB).
- Tier 1 Canada Research Chair on Systemic Autoimmune Rheumatic Diseases (to PRF).
- Canada Foundation for Innovation (CFI) – John R. Evans Leaders Fund (grant 34892 Microfluidic Imaging System, to CB).
- TAS and the FRQS fellowships (to MB and ET).

## Supplementary Material

Supplemental data

Supplemental video 1

Supplemental video 2

Supplemental video 3

Supplemental video 4

Supplemental video 5

Supplemental video 6

Supporting data values

## Version 1 12/02/2025

In-Press Preview

## Version 2 02/02/2026

Electronic publication
