# Supplementary material for: Immobilized IgG-containing immune complexes require platelets to recruit neutrophils during inflammation
Source: J Clin Invest. 2025 Dec 2;136(3):e195987. doi: 10.1172/JCI195987 (PMC12867151; doi:10.1172/JCI195987)
Supplement: Supplemental data [file jci-136-195987-s189.pdf]

Supplementary material

The recruitment of neutrophils by immobilized IgG-containing immune complexes critically requires platelets

Bellio, Allaeys et al.

Pages 1-7_ **Supplementary methods**
Pages 8-25_**Supplementary Figures**

SUPPLEMENTARY METHODS

Human platelet isolation

Venous blood from healthy volunteers or patients with arthritis was collected into BD Vacutainer® citrate tubes, kept at room temperature and centrifuged at 282 *g* for 10 min (no brake) at room temperature (RT). Supernatant (PRP) was collected, supplemented with 20% acid citrate dextrose (ACD) (45 mM sodium citrate, 25 mM citric acid, 82 mM dextrose (pH 4.5)) and 10 mM ethylenediaminetetraacetic acid (EDTA) and spun at 400 *g* for 2 min. Supernatant was spun for 5 min at 1100 *g* and the pellet was resuspended in 100 µL of Tyrode's buffer pH 6.5 (TB6.5) (135 mM NaCl, 3 mM KCl, 0.34 mM, Na_2HPO_4, 12 mM $NaHCO_3$, 20 mM Hepes, 0.5 mM $MgCl_2$, 5 mM glucose, 0.5 mg.mL^{-1} bovine serum albumin (BSA)) and transferred into 900 µL Tyrode's buffer pH 7.4 (TB7.4). Platelets were labelled for 1 hour with 2 µM CMRA (Invitrogen #C3451), spun at 1300 *g* for 5 min in the presence of 20% ACD and 10 mM EDTA, and resuspended in 80 µL of TB6.5 and 500 µL of TB7.4. Platelets were counted and diluted to 10^8 platelets.mL^{-1} in TB7.4.

Human neutrophil and monocyte isolation

Blood from healthy volunteers or patients with RA was collected in citrated tubes and 1 mM EDTA was added in whole blood. Neutrophils were isolated with STEMCELL EasySep™ Direct Human Neutrophil Isolation Kit (StemCell Technologies, #19666) and monocytes with STEMCELL EasySep™ Direct Human Monocyte Isolation Kit (StemCell Technologies, # 19669). Washed neutrophils (or monocytes) were spun at 300 *g* for 5 min, counted and diluted to 5 x 10^6 cells.mL^{-1} in TB7.4. Less than 1 platelet per 1,000 neutrophils was measured by flow cytometry (n=2) and 99.7% of neutrophils were exempt of platelets (mean ± standard deviation (99.7 ± 0.4%, n=8).

Human red blood cell isolation

Blood from healthy volunteers was collected in citrated tubes. 100 µl of red blood cell (RBC) fraction were collected after removing PRP/buffy coat and washed 5 times with 1.5 mL TB 7.4 (300 *g* centrifugation). RBC were counted and added to neutrophils at a ratio of 100 RBC for 1 neutrophil.

Mouse platelet isolation

Mice were anesthetized with 3% isoflurane in O_2. Blood (around 0.8 mL) was collected by cardiac puncture using a 1 mL syringe with a 25G needle containing 200 µL ACD. Blood was then transferred into 1.5 mL microcentrifuge tubes containing 350 µL TB6.5 and centrifuged at 400 *g* for 3 min before collecting the plasma and buffy coat. After adding 20% ACD and 10 mM EDTA, the supernatant was spun at 200 *g* for 2 min. The supernatant was collected and spun at 1100 *g* for 5 min, and the pellet was resuspended in 100 µL of TB6.5 and transferred into 700 µL TB7.4. Platelets were labelled for 1 hour with 2 µM CMRA (Invitrogen #C3451), then spun at 1300 *g* for 5 min in the presence of 20% ACD and 10 mM EDTA and resuspended in 100 µL of TB6.5 and 700 µL of TB7.4. Platelets were counted and diluted to 10^8 platelets.mL^{-1} in TB7.4.

Mouse whole blood microfluidic
Quadruple transgenic mice *FCGR2A^{TGN}::LY6gCre^{he}::Rosa26-TdT^{he}::CD41-YFP^{he}* expressing yellow platelets and red fluorescent neutrophils were anesthetized with 3% isoflurane in O_2. Blood (around 0.4 mL) was collected by cardiac puncture using a 1 mL syringe with a 25G needle containing 15 USP heparin units in 100µL of TB 6.5 for a final concentration of 30 USP units per mL. Microfluidic experiments were assessed within one hour of blood collection.

Mouse platelet depletion
Platelets from *FCGR2A^{TGN}* mice (healthy or arthritic) were depleted 24 to 48 h prior to blood collection, by intra-peritoneal injection (IP) of 2 µg.g^{-1} of rat anti-mouse GPIbα (Emfret Analytics, #R300), in order to efficiently isolate neutrophils from blood or proceed to the whole blood experiments in Bioflux. Isotype was used as control (Emfret Analytics, #C301).

Mouse neutrophil isolation
Mice were anesthetized with 3% isoflurane in O_2. Blood (around 0.8 mL) was collected by cardiac puncture using a 1 mL syringe with a 25G needle containing 200 µL of ACD and 2 mM EDTA. Red blood cells were lysed 5 min by ammonium-chloride-potassium (StemCell Technologies, #07850) and 2 mM EDTA at 4°C. Femurs were collected and flushed using a 1 mL insulin syringe with a 25G needle. Neutrophils from blood or bone marrow were isolated with STEMCELL EasySep™ Mouse Neutrophil Enrichment Kit (StemCell Technologies, #19762A). Washed neutrophils were spun at 300 *g* for 5 min, counted, and diluted to 5 x 10^6 neutrophils.mL^{-1} in TB7.4. Neutrophils were labelled for 30 min with 2 µM CMFDA (Invitrogen, #C7025), washed with TB7.4, and resuspended in TB7.4 at 10^6 neutrophils.mL^{-1}.

Platelet extracellular vesicle isolation
Platelets were diluted at 10^8 platelets/mL in Tyrode's Buffer pH 7.4 containing 5mM $CaCl_2$ and stimulated with thrombin (0.5 U.mL^{-1}; Millipore Sigma) for 18 h at room temperature. 10 mM EDTA was added to stop the stimulation. Remaining platelets were removed using a centrifugation step of 5 min at 1,300 *g*, and the supernatant was recovered. Platelet extracellular vesicles (PEV) contained in the supernatant

were enriched by ultracentrifugation at 18000 *g* for 60 min (18°C) and resuspended in filtered phosphate-buffered saline (PBS), pH 7.4. PEV were stained with a V450-conjugated anti-CD41 (Clone HIP8, **Supplemental Table 2**) and analyzed with a BD FACSymphony™ A1 with a Small Particle Detector. PEV size was characterized using fluorescent silica beads of known size (100, 500 and 1000 nm, Kisker Biotech, Germany). Protein content in PEV was measured using Pierce BCA Protein Assay Kit (ThermoFisherScientific, #23225) according to the manufacturer's protocol.

Platelet staining in microfluidic system

After adhesion, platelets were labelled in bioflux for 10 minutes using AF488 anti-CD62P (P-sel, **Supplemental Table 2**) or FITC Annexin V to label phosphatidylserine exposure (PS, BD Biosciences, #556420). Positive platelets for P-sel or PS were counted and expressed as % of adherent platelets (brightfield acquisition). PE anti-CD62P and FITC annexin V were used to evaluated double positive platelets.

Neutrophils in contact with platelet analysis (microfluidic system)

Platelets (10^8.mL^{-1} in TB7.4 containing 5mM $CaCl_2$) stained with CMRA (2 μM) were flowed 30 minutes on IC-coated capillaries. After a wash, Neutrophils (10^6.mL^{-1} in TB7.4 containing 5mM $CaCl_2$) stained with CMFDA were flowed 15 min into capillaries. Images of the same position (platelets: DsRed filter or neutrophils: FITC filter) were taken at 20X magnification and merged using Bioflux Montage software. Neutrophils in direct contact with platelets or in contact with IC only were counted and expressed as percentage of adhered neutrophils.

Flow cytometry analysis

After isolation, human and mouse neutrophils ($5x10^6$.mL^{-1}) were stained for 20 min at 4°C with specific antibodies (**Supplemental Table 2**) in PBS containing 0.5% BSA. Platelets (10^6.mL^{-1}) were stained 20 minutes at room temperature with specific antibodies (**Supplemental Table 2**) in TB7.4. Flow cytometry was performed using a BD Symphony A1 (BD Biosciences) for cells. All data were analyzed using FlowJo (v10.10.0). In order to collect infiltrating cells in joints, an incision was made on front paws and joints were washed with 200 μl of PBS containing 2mM EDTA and 0.5% BSA. Cells were spun 5 min at 300g and pellets were resuspended in 100 μl of PBS containing 2mM EDTA and 0.5% BSA and then stained with APC-Cy7 anti-mouse CD45 and APC-anti mouse Ly6G (details in **Supplemental Table 2**). FcyRIIA expression was evaluated on platelets and neutrophils in mouse whole blood. For neutrophils, the gating strategy involved gating first on total leukocytes (CD45 positive cells), then among leukocytes, the gate selected neutrophils (Ly6G positive cells). For platelets, the gating strategy involved gating FSC low CD41 positive cells. FcyRIIA mean intensity of fluorescence was measured on each population. Extracellular vesicles (EV) were analyzed with a BD FACSymphony™ A1 with a Small Particle Detector (BD Biosciences). Mouse PFP (3 μL) was stained in 50 μl with Vioblue anti-CD41, APC anti-CD62P and FITC-lactadherine **(Supplemental Table 2**) for 30 minutes at

room temperature. PBS (250 μL) was added and EV were evaluated by cytometry. Bioflux output containing human platelets and neutrophils was removed and were directly analyzed for EV content. 5 μL of output medium was stained in 50 μL with APC anti-CD62P and V450 anti-CD41. (**Supplemental Table 2**). PBS (250 μL) was added and EV were evaluated by cytometry. EV were quantified by the addition of a known concentration of fluorescent silica beads of 2 μm (Cy5, #Si2u-S5-1, Nanocs).

Platelet /neutrophil aggregates

Mouse blood was withdrawn through cardiac puncture and collected in 20% ACD, 35% Tyrode's buffer (pH 6.5). 5 μl of whole blood was diluted in 50 μl of PBS for 5 minutes and then stained at room temperature for 20 minutes, using APC anti-Ly6G, BV421 anti-CD41 and APC-Cy7 anti-CD45 (details in **Supplemental Table 2**) to detect platelet/neutrophil aggregates. 500 μL of PBS was added prior to flow cytometry analyses and 500 to 1,000 CD45+Ly6G+ cells were acquired. The gating strategy involved gating first on total leukocytes (CD45 positive cells), then among leukocytes, the gate selected neutrophils (Ly6G positive cells) and then the percentage of CD41 positive cells in neutrophil subpopulation was measured and defined as platelet/neutrophil aggregates.

NET detection

MPO-DNA complexes were quantified by ELISA as followed: an anti-MPO antibody (2 μg.mL^{-1}; clone 4A4, Bio-Rad, #0400-0002) or mIgG2b isotype (2 μg.mL^{-1}; as control) were coated in PBS overnight at 4°C, washed 5 times and then blocked with 2% BSA in PBS for 2 hours at room temperature. The plate was washed 5 times before incubation for 90 minutes at room temperature with 5% mouse plasma or 25% microfluidic-output in blocking buffer. Plate was washed 5 times, and then incubated for 90 minutes at room temperature with anti-DNA antibody (1:12; Cell Death Detection ELISA, Millipore Sigma, #11544675001). After 5 washes, the plate was developed with TMB substrate (ThermoFisher Scientific, #N301). NETs are expressed as MPO/DNA_OD_{450nm} minus mIgG2b/DNA_OD_{450nm}: Delta OD_{450nm}.

Calcium mobilization

Neutrophils were stained 30 min at room temperature with FLUO-4 (1μM), Invitrogen #F14217) and then flowed into microfluidic capillaries. Neutrophil calcium signaling was visualized by FLUO-4 fluorescence acquisition at 100X magnification (FITC settings) every second for 3 min.

Mass cytometry CyTOF®

The Maxpar® Direct™ Immune Profiling System (Standard BioTools) was used with blood from healthy volunteers or patients with rheumatoid arthritis (diagnosis within 6 months) according to the manufacturer's instructions. Briefly, heparin was added (100 U.mL^{-1}) into the blood for 20 min at room temperature (RT). 270 μL of heparinized blood was added directly in tubes containing dry antibodies (30-marker panel). Blood was stained for 30 min at RT and then, red blood cells were

lysed using 250 µL of Cal-Lyse for 10 min at RT and followed by the addition of 3 ml of Maxpar water and an additional 10 min of incubation. The tubes were washed 3 times in Maxpar Cell Staining Buffer followed by fixation in 1.6% paraformaldehyde for 10 min. After fixation, the cells were spun to a pellet, the fixative removed, and the pellet was resuspended in 1 ml of the 125 nm Cell-ID™ Intercalator-Ir and incubated overnight at 4°C. Cells were washed in Cell Acquisition Solution and resuspended at 10^6 cells.mL^{-1}. Samples were acquired on a Helios® mass cytometer (Standard BioTools). Setup and tuning were performed according to the manufacturer's protocol; cells were acquired with a speed of 250-400 events/second. After acquisition, data were normalized using the CyTOF Software, cleaned up following manufacturer's instructions and analyzed with Flowjo (v10.10.0).

K/BxN serum transferred arthritis

Arthritis was induced in male mice (8–12 weeks old) by intraperitoneal (IP) injections of arthritogenic K/BxN serum (150 µL) performed on days 0 and 2 of the experiment. Control antibody (anti-HRP BioXCell #BE0088 or anti-KLH BioXCell#BE0090), blocking PSGL-1 antibody (clone 4RA10, BioXCell #BE0186) or blocking CD11b antibody (clone M1/70, BioXcell#BE0007) were IP injected in mice (100 µg per mouse) every other day beginning on day 0 for a prophylactic treatment, and every day beginning day 3 for a therapeutic treatment. We took daily measurements of ankle thickness (measure of the malleoli using spring-loaded dial calipers (Newman Tools Inc) with the ankle in a fully flexed position), which correlates with disease severity, along with a clinical index graded on a scale 0–12.

Histology

Arthritic joints were collected 7 days after the first K/BxN serum injection, fixed 48 h in 4% paraformaldehyde (PFA), washed in PBS and decalcified for 30 days in 14% EDTA at 4°C (solution changed every 2–3 days). Tissues were then dehydrated, embedded in paraffin, sectioned at 10 µm thickness, and stained with hematoxylin and eosin (H&E). Histological scoring (based on)(122) was performed with blinding to prevent bias and expressed as inflammation score, graded on a scale 0–5 with respect to cell infiltration and cartilage/bone erosion. 0: Normal; 1: Minimal cell infiltration, minimal loss of cartilage (no chondrocyte loss); 2: Mild infiltration, mild loss of cartilage (superficial chondrocyte loss); 3: Moderate infiltration, moderate loss of cartilage (moderate multifocal chondrocyte loss); 4: Marked infiltration, marked loss of cartilage (marked multifocal chondrocyte loss); 5: Severe infiltration, severe diffuse loss of cartilage (severe multifocal chondrocyte loss).

Joint immunofluorescence staining

Arthritic joints were collected 7 days after the first K/BxN serum injection, fixed 48 h in 4% paraformaldehyde (PFA), washed in PBS and decalcified for 30 days in 14% EDTA at 4 degrees (solution changed every 2–3 days). Antigen retrieval was performed on 10 µm frozen sections using Diva Decloaker (Biocare Medical,

#DV2005L2J) for 20 min at 95°C and sections were blocked 1 hour with PBS-Tween solution containing 10% normal goat serum (JIR #017-000-121). Joint sections were stained overnight at 4°C with anti-endomucin (10 μg.mL^{-1}, Rat anti-mouse, SantaCruz Biotechnology #sc-65495) or with irrelevant antibodies. Sections were washed and incubated with goat anti-rat AF568 (2 μg.mL^{-1}, Invitrogen #A-11077) and donkey anti-mouse-IgG AF647 (2 μg.mL^{-1}, JIR # 715-605-151) for 90 minutes at RT. Nuclei were stained using Hoechst (10 μg mL^{-1}, Invitrogen #H3570) for 5 min and slides were mounted using Dako fluorescence mounting medium (Agilent Technologies, #S3023). To measure leukocyte infiltration, sections were stained with rat anti-mouse CD45 (10 μg.mL^{-1}, BD #553076) 18h at 4°C and after 3 washes, section were incubated with goat anti-rat AF568 (2 μg.mL^{-1}, Invitrogen #A-11077). Nuclei were stained using Hoechst (10 μg mL^{-1}, Invitrogen #H3570) for 5 min and slides were mounted using Dako fluorescence mounting medium (Agilent Technologies, #S3023). Images were acquired using a Z2 confocal microscope with a LSM 800 scanning system (Zeiss, Germany) and a 20X or a 40X oil objective (Apochoromat/1.4, Zeiss). Images were processed using ZEN 3.11 software (Zeiss) and Z-stack projections of 3 μm in total thickness are represented. 6 to 8 random fields (around synovium) were acquired in order to evaluate leukocyte infiltration.

Bone marrow chimera
Fifteen million bone marrow cells from *FCGR2A^{TGN};P-sel$^{+/+}$* mice (6–8 weeks) were intravenously (iv) injected into lethally irradiated (1,021 cGy, Gammacell 40 irradiator, Nordion) into FcγRIIA^{Null};P-sel$^{+/+}$ or FcγRIIA^{Null};P-sel$^{-/-}$ recipients in phosphate buffered saline. Mice were under antibiotic treatment (sulfamethoxazole (1mg/mL) and trimethoprim (0.2mg/mL) for one week before irradiation and 4 weeks after. Successful engraftment was verified by measuring FcγRIIA expression on platelets by flow cytometry using FITC anti-FcγRIIA (**Supplemental Table S2**). Absence of P-selectin on platelets from P-sel deficient mice was confirmed by flow cytometry on isolated-platelets stimulated 15 min with thrombin (0.5 U.mL^{-1}) and using FITC anti-CD62P and PE anti-activated αIIbβ3 (**Supplemental Table S2**).

Intravital microscopy
Three days after the first K/BxN serum injection, platelet and neutrophil circulation in the vasculature of the joint were examined using high-speed widefield fluorescence microscopy. Control antibody (anti-HRP) or blocking PSGL-1 antibody (clone 4RA10) were IP injected in mice (100 μg per mouse) every day since day 0. All videos were acquired with an Olympus BX51WI workstation equipped with a Fluorescence Excitation Illumination System (Lumen 300-LED, Prior Scientific) using an Olympus 20X or 40X water objective with a numerical aperture of 0.50. The microscope was equipped with a dual emission image splitter (Optosplit II system) and a fast acquisition camera (Digital CMOS camera ORCA-Flash4.0 V2, C11440-22CU, Hamamatsu) that permitted a high capture rate of 100 frames per second. Nirvana software (BliQ Photonics) was used to acquire the videos. Prior to the intravital imaging session, mice were anesthetized with 2%

isoflurane (vol/vol) with oxygen (1 $L.min^{-1}$) and then placed under the microscope. A heat-pad was used to maintain the body temperature of the mice. Ophthalmic ointment was applied to avoid ocular dryness. Different regions in the joint were recorded for 40 seconds for a total of 3 regions on each ankle. Blinded analyses were performed on 3 regions for each mouse using ImageJ. Neutrophils were evaluated frame by frame with manual tracking based on fluorescence (red channel) and size (8 to 12 μm). Circulating neutrophils were defined as fast cells, observed at different locations in 2 subsequent frames; adherent neutrophils as cells observed at the same location in 300 subsequent frames; rolling neutrophils as slowly moving cells observed at the same location in 2 subsequent frames and platelet-neutrophils as neutrophils directly interacting with the same platelet (turquoise) in at least 2 subsequent frames.

Data and reagent availability

All primary data and reagents can be made available on reasonable request. Mouse strains must be shared in agreement with institutional MTA.

Figure schematics were created in https://BioRender.com

Mice

$FCGR2A^{Null}$ (C57BL/6J, strain#000664), $FCGR2A^{TGN}$ (hemizygous (he), strain#003542), $Fcgrt^{-/-}$ (strain#003982), $Fcgr2b^{-/-}$ (strain#002848), $FcRg^{-/-}$ (strain#017793) and P-$selectin^{-/-}$ (strain#002217) mice were purchased from The Jackson Laboratory. $FCGR2A^{TGN}$ mice express the human FcγRIIA on platelets, megakaryocytes, monocytes, macrophages, neutrophils, eosinophils, basophils, mast cells, and dendritic cells.(56) FcγRIIA expression was evaluated on platelets and neutrophils (**Supplemental Figure S15**). For breeding, $FCGR2A^{TGN}$ in a complete C57Bl6/J background with a hemizygous expression of FcγRIIA are bred with wild type C57BL/6J, thereby generating $FCGR2A^{TGN}$ and the controls $FCGR2A^{Null}$. The $Itgb3^{-/-}$ mice,(123) $Tph1^{-/-}$ mice,(63) and CD41-YFP mice(124) were crossed with $FCGR2A^{TGN}$ mice to obtain $FCGR2A^{TGN}$;$\beta3^{-/-}$, $FCGR2A^{TGN}$;$Tph1^{-/-}$, and $FCGR2A^{TGN}$;CD41-YFP mice. $FCGR2A^{TGN}$::CD41-YFP^{he} mice were generated by crossing $FCGR2A^{TGN}$ mice with mice expressing yellow fluorescent platelets (CD41-YFP^{he}). Then the quadruple transgenic mice $FCGR2A^{TGN}$::$LY6gCre^{he}$::Rosa26-TdT^{he}::CD41-YFP^{he} were obtained by crossing a male $FCGR2A^{TGN}$::CD41-YFP^{ho} with a female $LY6gCre^{ho}$::Rosa26-TdT^{ho} expressing red fluorescent neutrophils. $Mpig6b^{-/-}$ and $Treml1^{-/-}$ C57BL6J mice were obtained from V. Washington and Y. Senis, and crossed with $FCGR2A^{TGN}$ mice. Mice were backcrossed to C57BL/6J background except for $Fcgr2b^{-/-}$.

SUPPLEMENTARY FIGURES

The recruitment of neutrophils by immobilized IgG-containing immune complexes critically requires platelets
Bellio, Allaeys et al.

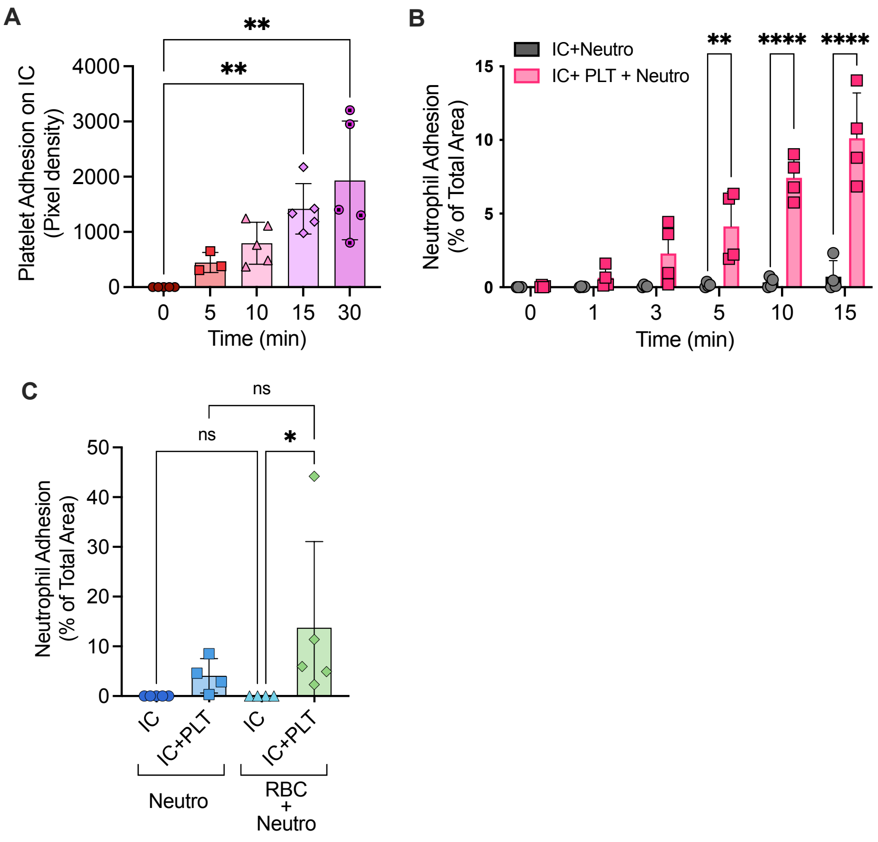

Figure S1: Platelet and neutrophil adhesion on IC. Human platelets (PLT) and neutrophils were isolated from the blood of healthy volunteers. Platelets (10^8 platelets.mL^{-1} labelled with CMRA) or control solution were perfused in microcapillary coated with human immune complexes (IC, 500 μg.mL^{-1}) for 30 minutes at a shear stress of 2 dyn.cm^{-2} in Bioflux. After washing the microcapillaries, neutrophils (10^6 neutrophils.mL^{-1} labelled with CMFDA) were perfused for 15 minutes. Platelet **(A)** or neutrophil **(B)** adhesion was quantified overtime and expressed as mean ± standard deviation (SD) (n=3-5), **P < 0.01, ****P < 0.0001, (A) Kruskal-Wallis with Dunn's multiple comparisons test, (B) Two-way ANOVA with Šídák's multiple comparison test. **(C)** Neutrophils (Neutro) were perfused with or without Red Blood Cells (RBC: 10^8 cells.mL^{-1}) for 15 minutes on IC or IC with PLT. Neutrophil adhesion was measured at 15 minutes and expressed as mean ± SD (n=4-5), Kruskal-Wallis with Dunn's multiple comparisons test, *P < 0.01 (ns: not significant).

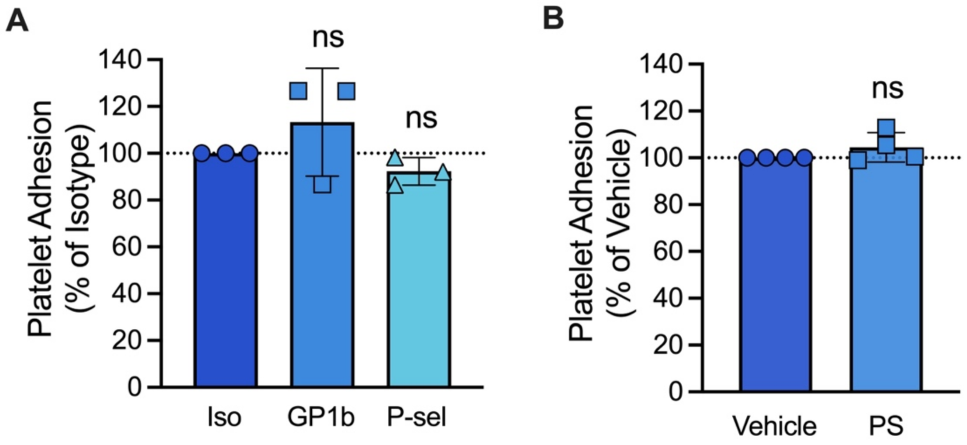

Figure S2: Platelet adhesion on IC in presence of blocking antibodies. (A and B) Platelets were isolated from human blood (10^8 platelets.mL^{-1} labelled with CMRA) and perfused in microcapillary coated with human immune complexes (IC, 500 μg.mL^{-1}) for 30 minutes at a shear rate of 2 dyn.cm^{-2} in Bioflux. Microcapillaries were washed for 20 minutes, in presence of **(A)** isotypic control antibodies (Iso), blocking antibodies against GP1b, P-sel, or **(B)** Annexin V (PS) and platelet adhesion was quantified. Data are expressed as mean ± SD (n=3-4), **(A)** Kruskal-Wallis test with Dunn's multiple comparisons test. **(B)** Wilcoxon matched-pairs signed rank test (ns: not significant).

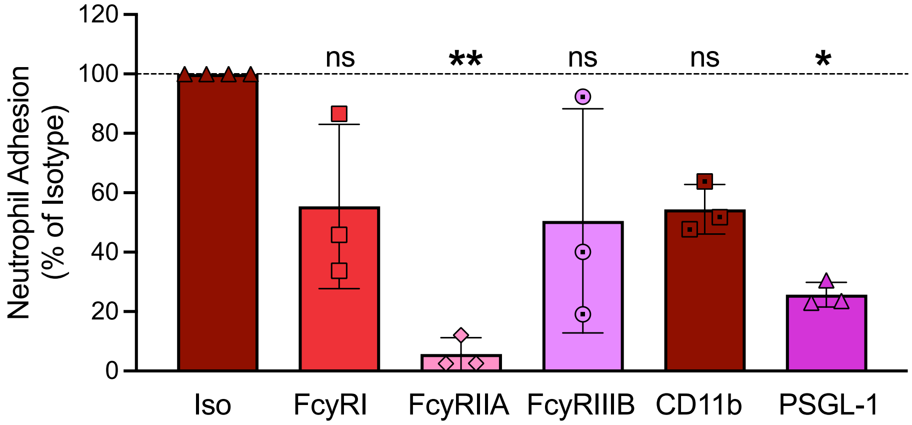

Figure S3: Neutrophil adhesion at 1 dyn.cm^{-2} in presence of blocking antibodies. Platelets were perfused for 30 min on IC, capillaries were washed and neutrophils were perfused for 15 min in presence of a control antibody (Iso) or blocking antibody directed against FcyRI (10 μg.mL^{-1}), FcyRIIA (5 μg.mL^{-1}), FcyRIIIB (10 μg.mL^{-1}), CD11b (10 μg.mL^{-1}) or PSGL-1 (10 μg.mL^{-1}). Area covered by neutrophils at 15 min was quantified and expressed as percentage of isotype control, mean ± standard deviation (SD) (n = 3-4), * $P < 0.05$ and **$P < 0.01$, Kruskal-Wallis with Dunn's multiple comparisons test (ns: not significant).

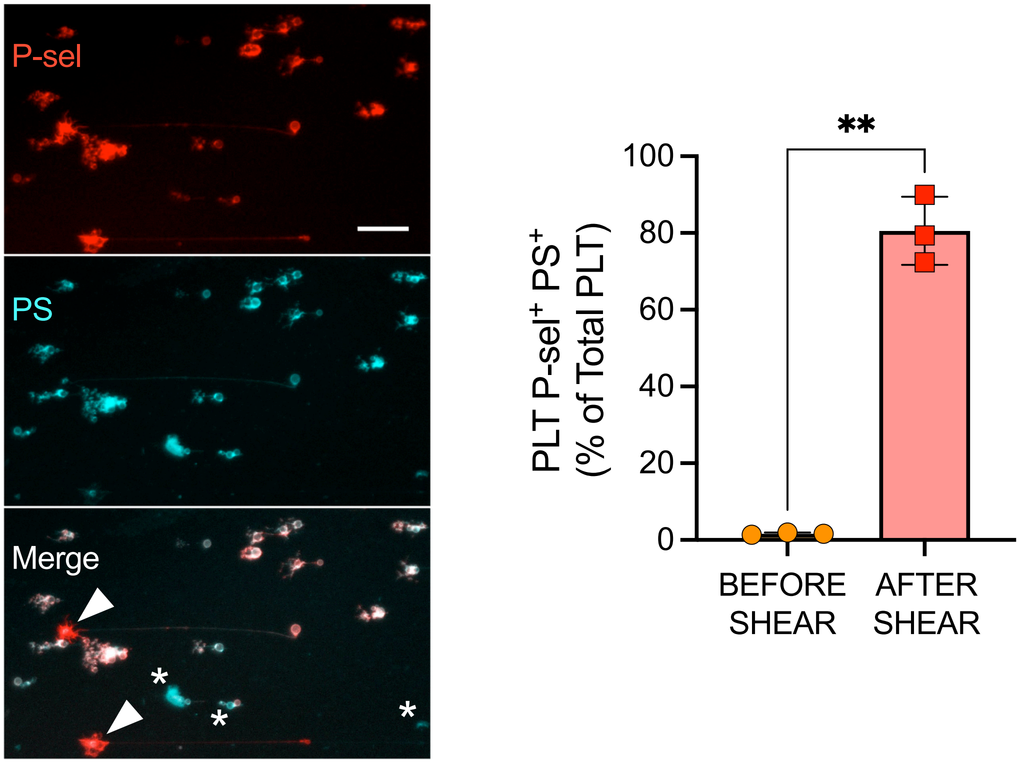

Figure S4: P-selectin and phosphatidylserine expression by platelets after shear stress on immobilized IC. Platelets were isolated from healthy volunteers and perfused (10^8.mL^{-1}) into capillaries coated with IC (500 μg.mL^{-1}) for 30 minutes at 2 dyn.cm^{-2}. Capillaries were washed, PE-anti CD62P (P-sel, 100 μL.mL^{-1}) and then FITC-Annexin V (PS, 25 μL.mL^{-1}) were perfused for 10 minutes each. Left panel, representative images after IC/Shear (scale bar 10μm), platelets expressing only P-selectin (P-sel, red) or platelets expressing only phosphatidylserine (PS, cyan) are indicated with arrowheads and asterisks respectively. Right panel, graphic representing percentage of platelets expressing both P-sel and PS. Data are expressed as mean ± SD (n = 3), **$P < 0.01$, unpaired t test with Welch's correction.

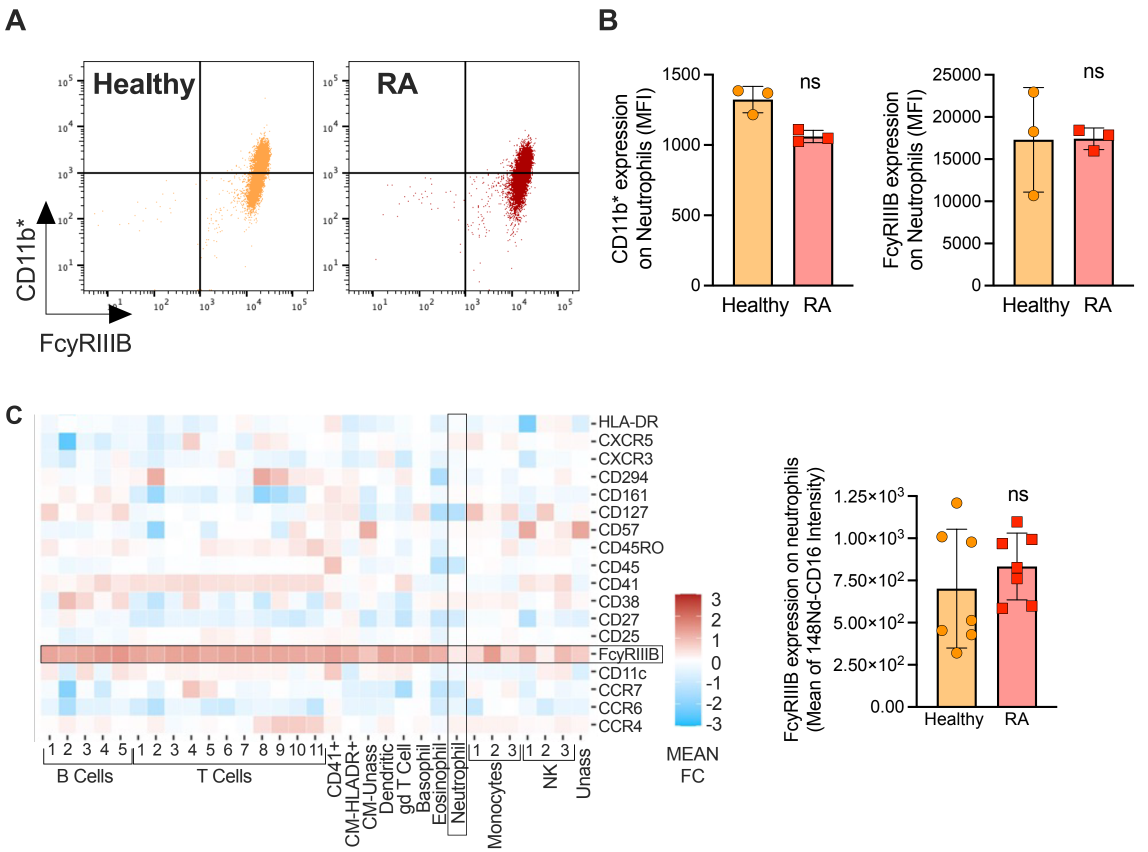

Figure S5: Expression of CD16 and activated form of CD11b on neutrophils. Human neutrophils were analyzed by flow cytometry and CyTOF. **A)** Representative dot plots of CD16 and activated form of CD11b (CD11b*) expression on neutrophils from heathy volunteer (left panel) or RA patient (right panel). **B)** Mean fluorescence intensity (MFI) quantifications are represented for CD11b* (left panel) and FcγRIIIB (right panel). Data are expressed as mean ± standard deviation (SD) (n=3), Mann Whitney test, ns: not significant. **(C)** CyTOF analysis identified immune cell clusters in peripheral blood of healthy volunteers and RA patients. Left panel: heatmap showing the mean fold-change variation (mean FC) of different expression markers in RA patients relative to healthy volunteers (n = 4–5). Red: positive fold change; blue: negative fold change. Cell clusters are assigned as: B Cells #1(CD27$^-$), B Cells #2 (CD27$^+$_unassigned), B Cells #3 (Non-Switched Memory), B Cells #4 (Plasmablast), B Cells #5 (Switched Memory), T Cells #1(CD4$^-$CD8$^+$), T Cells #2 (CD4$^+$CD8$^+$), T Cells #3 (CD4$^+$ Central Memory), T Cells #4 (CD4$^+$ Effector Memory), T Cells #5 (CD4$^+$ EMRA), T Cells #6 (CD4$^+$ Naive), T Cells #7 (CD4$^+$ Treg), T Cells #8 (CD8$^+$ Central Memory), T Cells #9 (CD8$^+$ Effector Memory), T Cells #10 (CD8$^+$ EMRA),T Cells #11 (CD8$^+$ Naive), Monocytes #1 (CD14$^-$CD16$^+$), Monocytes #2 (CD14$^+$CD16$^-$), Monocytes #3 (CD14$^+$CD16$^+$), Natural Killer (NK) #1 (CD56$^+$CD16$^-$), NK #2 (CD56$^+$CD16$^+$), NK #3 (NKT) or root unassigned (Unass). Right panel, barplot of neutrophil FcγRIIIB expression (Mean of 148Nd-CD16 intensity). Data are expressed as mean ± SD (n=7), Mann-Whitney test. (ns = not significant).

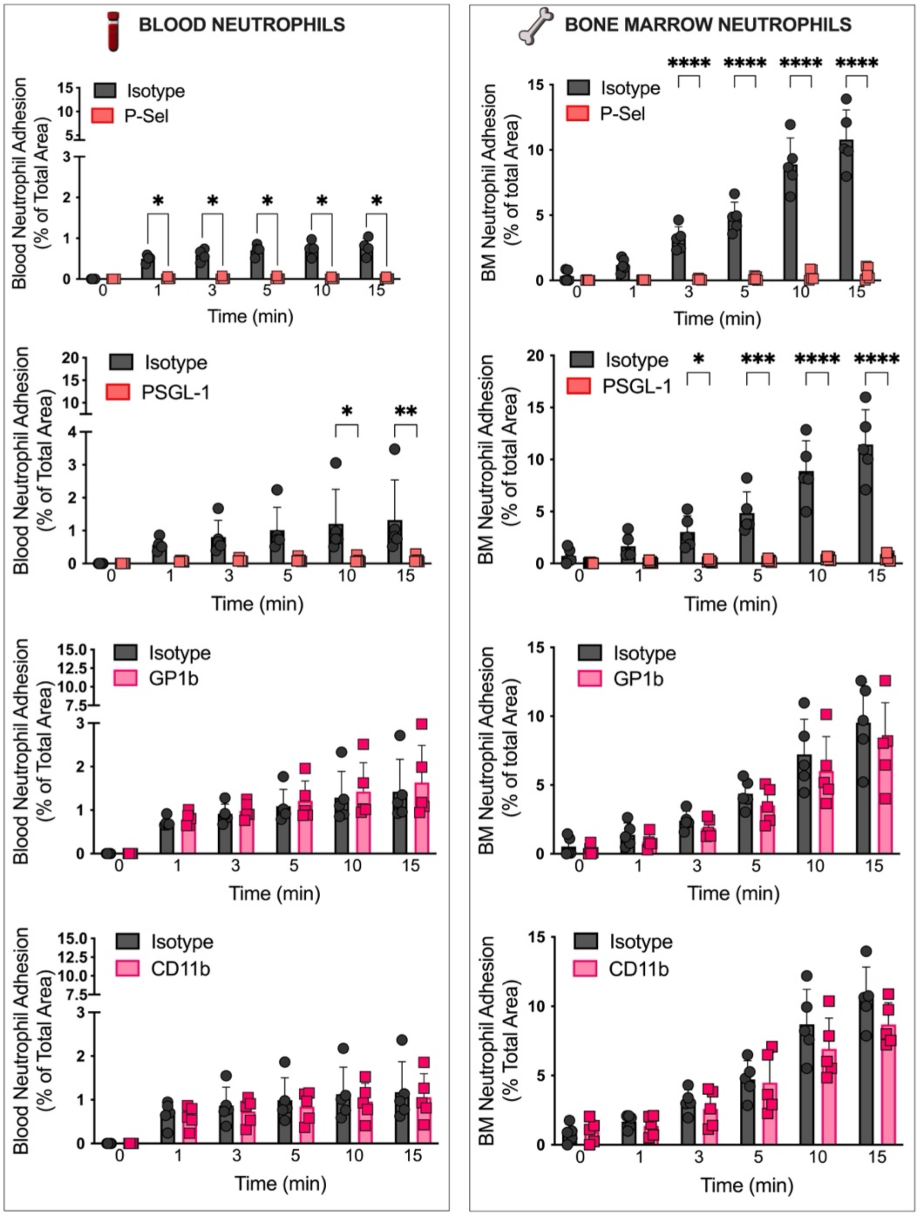

Figure S6: Comparison of blood and bone marrow neutrophil adhesion. Platelets and neutrophils, either from blood or bone marrow (BM), were isolated from FcγRIIA^{TGN} mice. Platelets (10^8 platelets.mL^{-1} labelled with CMRA) were perfused in microcapillary coated with mouse immune complexes (500 μg.mL^{-1}) for 30 minutes at a shear stress of 2 dyn.cm^{-2} in Bioflux. Microcapillaries were washed in presence of control antibody or platelet blocking antibody directed against P-selectin (P-sel) and GP1b. Neutrophils (10^6 neutrophils.mL^{-1} labelled with CMFDA) isolated from blood (left panels) or bone marrow (BM, right panels) were perfused for 15 minutes in presence of control antibody (Isotype) or blocking antibody directed against PSGL-1, CD11b, P-sel or GP1b. Neutrophil adhesion was measured overtime and data were expressed as mean ± SD (n=5), *P < 0.05, **P < 0.01, ***P < 0.001 and ****P < 0.0001, two-way ANOVA with Šídák's multiple comparisons test.

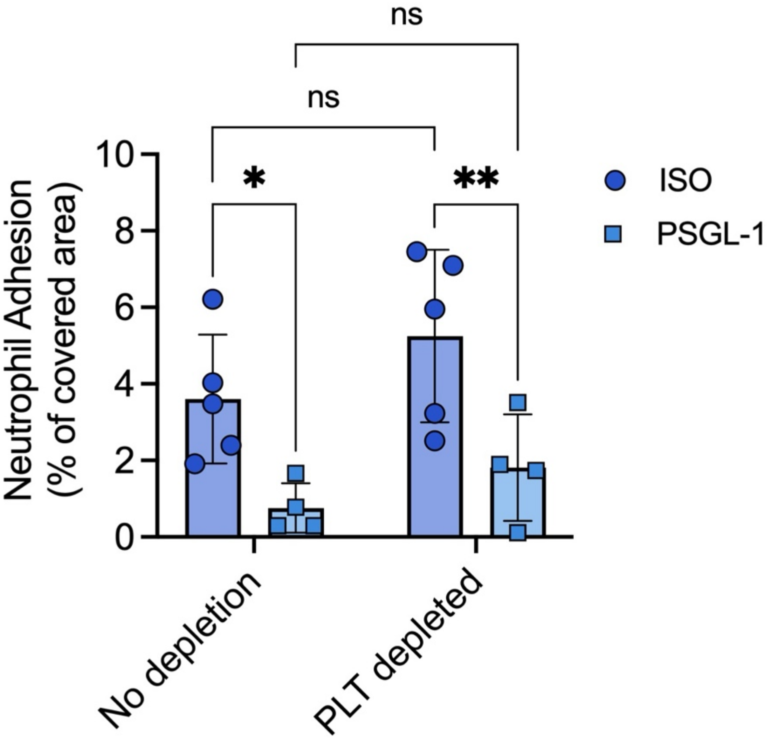

Figure S7: Effect of platelet depletion on neutrophil adhesion on ICs. Platelets from arthritic mice expressing FcγRIIA (10^8 platelets.mL^{-1} labelled with CMRA) were perfused in microcapillary coated with murine immune complexes (500 μg.mL^{-1}) for 30 minutes at a shear stress of 2 dyn.cm^{-2} in Bioflux. After washing the microcapillaries**,** neutrophils from arthritic mice expressing FcγRIIA (10^6 neutrophils.mL^{-1} labelled with CMFDA) depleted or not in platelets were then perfused with isotype (ISO) or anti-PSGL-1 (10 μg.mL^{-1}) for 15 minutes. Neutrophil adhesion was quantified and expressed as mean ± SD (n=4-5), *P < 0.05 and ***P < 0.01, two-way ANOVA with Uncorrected Fisher's LSD (ns: not significant).

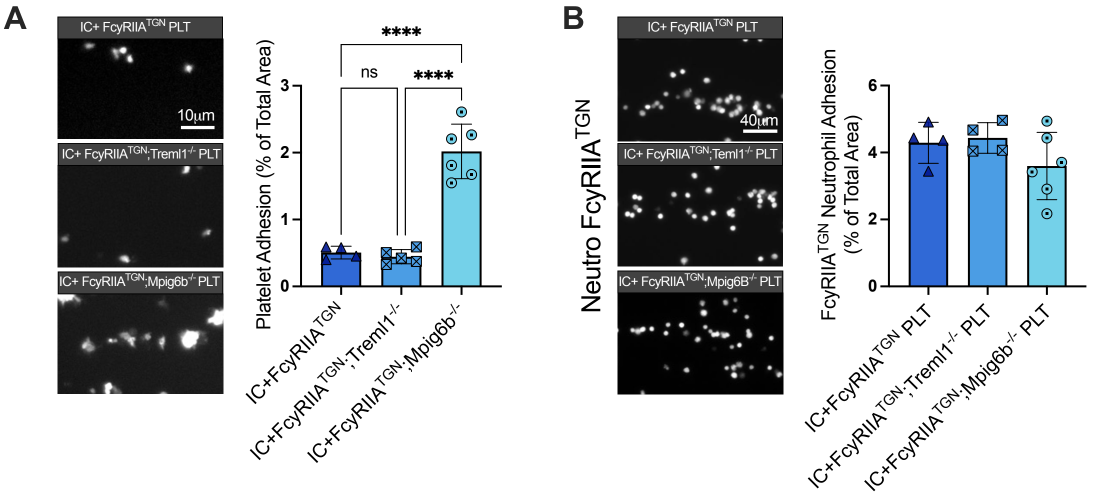

Figure S8: Role of platelet ITIMs on neutrophil adhesion to immune complexes. (A–B) Platelets were isolated from mice deficient for immunoreceptor tyrosine-based inhibitory motif (ITIM) receptor TLT1 and G6bB but expressing FcγRIIA (FcγRIIA^{TGN};Treml1$^{-/-}$ and FcγRIIA^{TGN};Mpig6b$^{-/-}$). **(A)** Representative images (left panel) and quantification (right panel) of platelet adhesion on IC. **(B)** Representative images (left panel) and quantification (right panel) of neutrophil (expressing FcyRIIA) adhesion on IC with FcγRIIA^{TGN} platelets expressing or not ITIM receptors. Data are expressed as mean ± SD (n = 4–6), ****$P < 0.0001$, one-way ANOVA with Tukey's multiple comparisons.

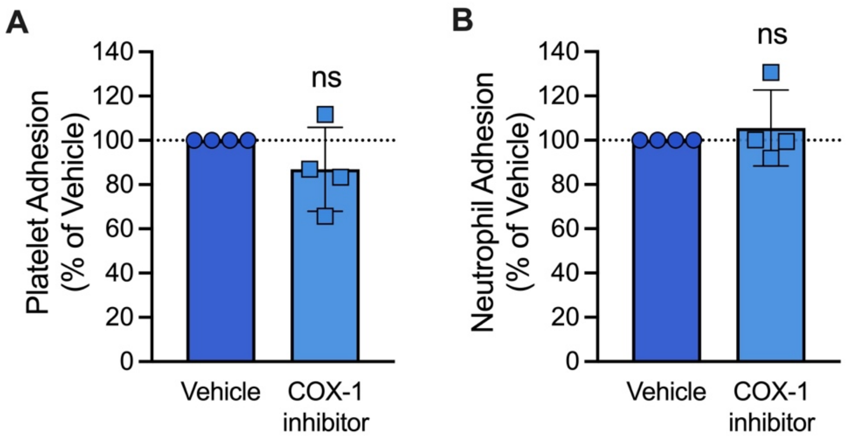

Figure S9: Effect of COX-1 inhibitor. Human platelets and neutrophils were isolated from the blood of healthy volunteers. **(A)** Platelets (10^8 platelets.mL^{-1} labelled with CMRA) were perfused in microcapillary coated with human immune complexes (500 μg.mL^{-1}) for 30 minutes at a shear stress of 2 dyn.cm^{-2} in Bioflux. After washing the microcapillaries in presence of COX-1 inhibitor (SC-560, 100nM) or vehicle (DMSO). **(B)** Neutrophils (10^6 neutrophils.mL^{-1} labelled with CMFDA) were then perfused with or without COX-1 inhibitor for 15 minutes. Platelet and neutrophil adhesion were quantified and expressed as mean ± SD (n=4), Wilcoxon matched-pairs signed rank test (ns: not significant).

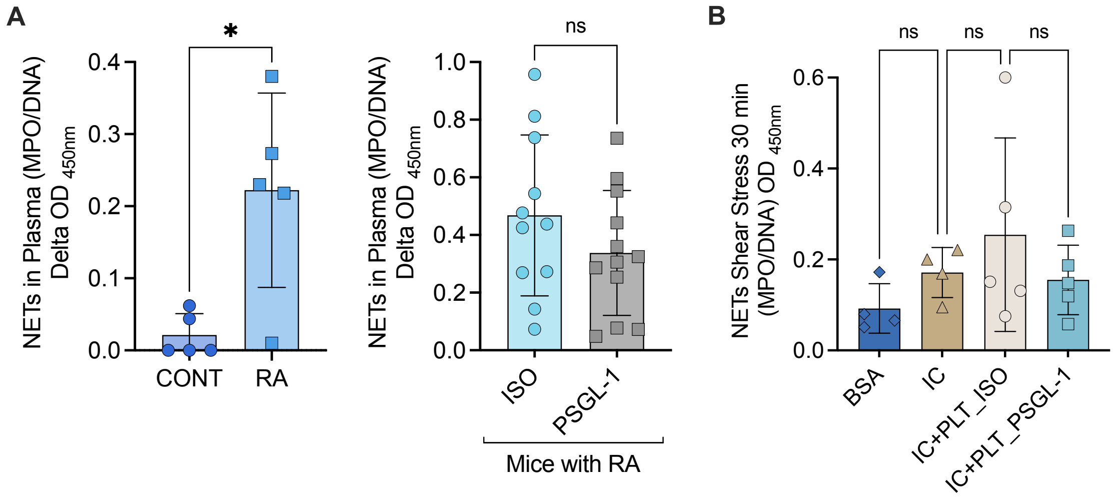

Figure S10: NET formation in course of arthritis or neutrophil recruitment on IC. (A) NETs (MPO/DNA complexes) were measured in plasma from control FcγRIIA^{TGN} mice (CONT) and arthritic FcγRIIA^{TGN} mice (RA) (left panel) or in mice that received a prophylactic treatment of anti-PSGL-1 or Isotype (ISO) (right panel). Data are expressed as mean of delta OD_{450nm} (MPO/DNA minus isotype/DNA OD_{450nm}) ± SD (n=5-12), *$P < 0.05$, Mann-Whitney test (ns: not significant). **(B)** NETs were evaluated in microfluidic outputs after a shear stress of 2 $dyn.cm^{-2}$. Neutrophils were run 30 minutes on BSA (control), immune complexes (IC), IC + platelets (PLT) with isotype (ISO) or IC + PLT with anti-PSGL-1. Data are expressed as mean OD_{450nm} (MPO/DNA) ± SD (n=4-5), Kruskal-Wallis with Dunn's multiple comparisons test. ns: not significant.

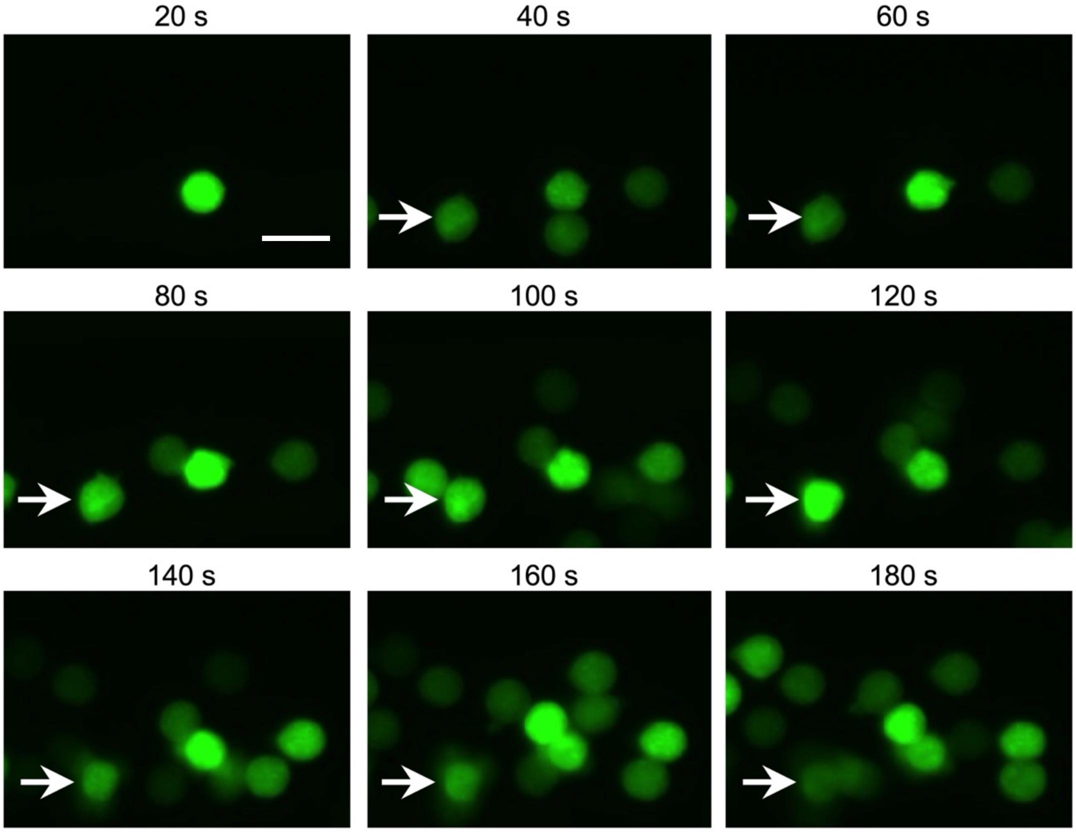

Figure S11: Neutrophil adhesion on IC with platelets induces calcium mobilization. Platelets (10^8 platelets.mL^{-1} labelled with CMRA) were perfused in microcapillaries coated with human immune complexes (500 μg.mL^{-1}) for 30 minutes at a shear stress of 2 dyn.cm^{-2} in Bioflux. Capillaries were washed and neutrophils (10^6 neutrophils.mL^{-1} labelled with FLUO-4) were perfused for 3 minutes. Representative images (n=3), every 20 seconds of perfusion, showing pulsing calcium mobilization. An adherent neutrophil with calcium pulse is indicated with an arrow (scale bar = 20 μm).

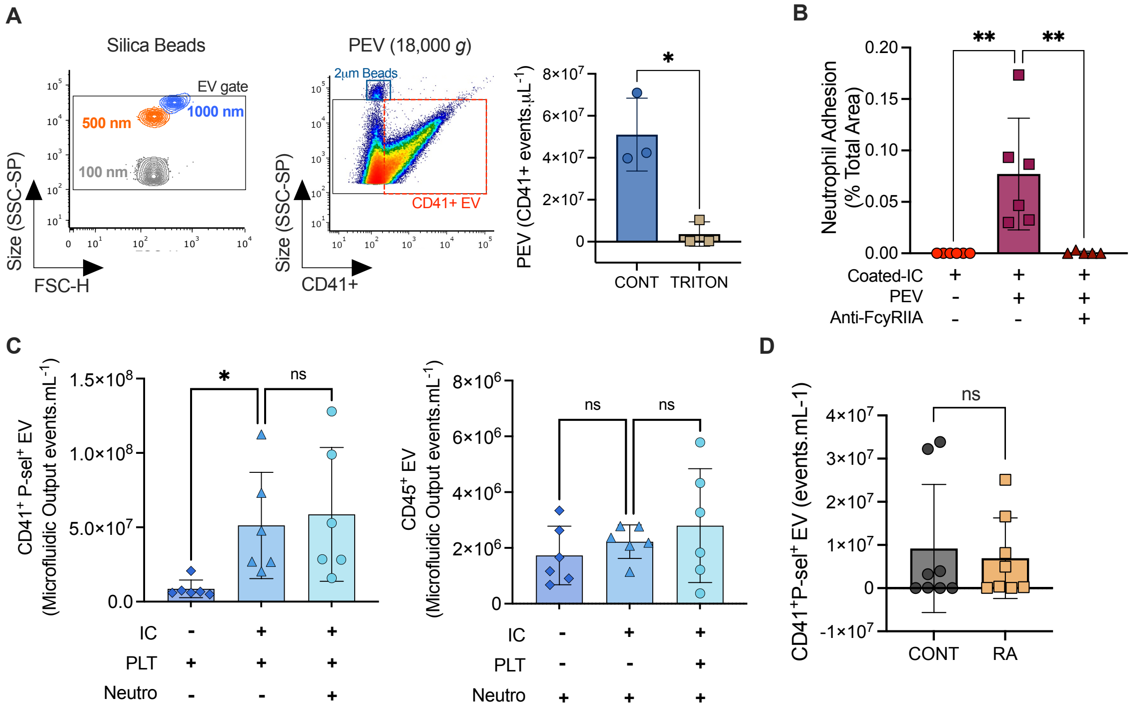

Figure S12: Platelet extracellular vesicles induce neutrophil adhesion on IC. (A) Purified platelet extracellular vesicles (PEV, 18,000 *g* pellet) were analyzed by flow cytometry. An EV gate was calibrated on silica beads size (left panel). Detergent lysis (TRITON, mid panel) was used to confirm EV lipid membrane content. Data are expressed as mean ± standard deviation (SD) (n=3), paired T-test, * $P < 0.05$. **(B)** Purified PEV (150 μg.mL^{-1}) were perfused in microcapillaries coated with human immune complexes (IC, 500 μg.mL^{-1}) for 30 minutes at a shear stress of 2 dyn.cm^{-2} in Bioflux. After washing the microcapillaries, neutrophils (10^6 neutrophils.mL^{-1} labelled with CMFDA) were perfused for 15 minutes in presence of control antibody or blocking FcγRIIA antibody. Neutrophil adhesion was quantified at 15 minutes and expressed as mean ± SD (n=5-6), *$P < 0.05$ and **$P < 0.01$, Šídák's multiple comparisons test. **(C)** PEV (CD41$^+$P-sel$^+$ EV) or neutrophil EV (CD45+) generation in microfluidic output were analyzed by flow cytometry. Data are expressed as mean ± SD (n=6), *$P < 0.05$, Kruskal-Wallis with Dunn's multiple comparisons test. **(D)** PEV (CD41$^+$P-sel$^+$ EV) were measured in plasma from arthritic mice (RA) or control mice (CONT) and expressed as mean ± SD, Mann-Whitney test, n=8 (ns=not significant).

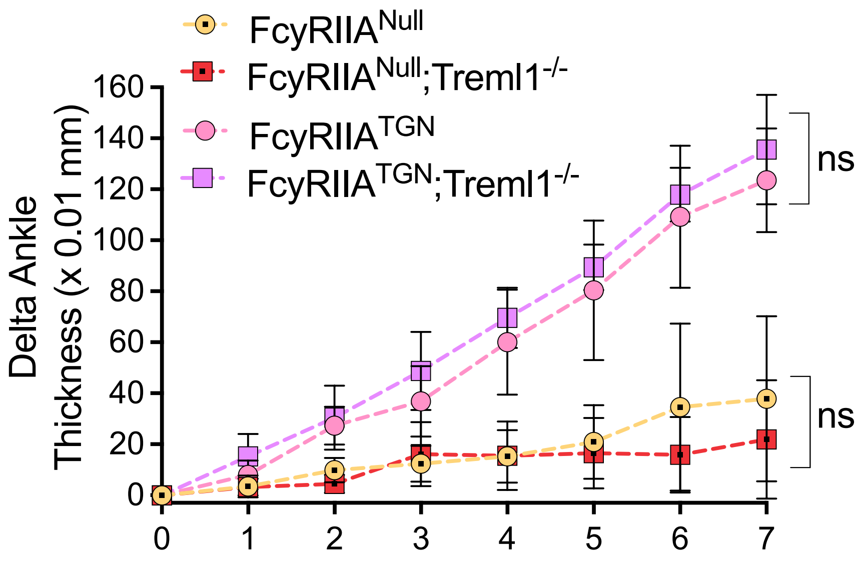

Figure S13: Arthritis in Treml1 deficient mice. Arthritis was induced with two intraperitoneal (IP) injections (2 days apart) of K/BxN serum in FcγRIIA^{Null} (orange) FcγRIIA^{Null};Treml1$^{-/-}$ (red), *FcγRIIA^{TGN}* (pink) or FcγRIIA^{TGN};Treml1$^{-/-}$ (purple) mice. Disease severity was monitored daily by measuring ankle thickness. Data are expressed as mean ± standard deviation (SD) of delta ankle thickness (n = 5–8 per group), Mixed-effect analysis with Tuckey's multiple comparison test between FcγRIIA^{Null} and FcγRIIA^{Null};Treml1$^{-/-}$ or FcγRIIA^{TGN} and FcγRIIA^{TGN};Treml1$^{-/-}$ mice.

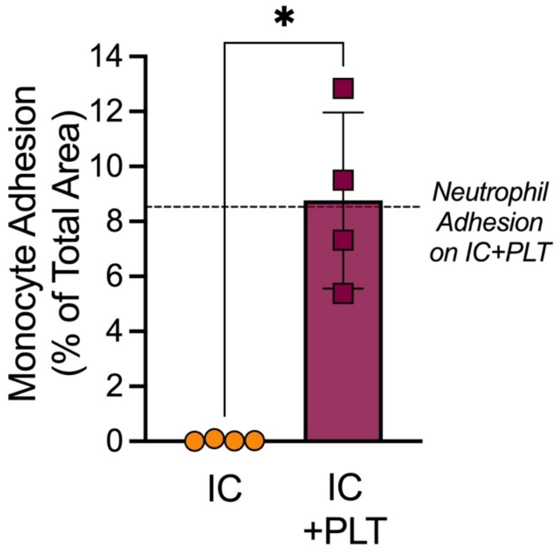

Figure S14: Monocyte adhesion on IC in presence of platelets. Human platelets (PLT) and monocytes were isolated from the blood of healthy volunteers. Platelets (10^8 platelets.mL^{-1} labelled with CMRA) or control solution were perfused in microcapillary coated with human immune complexes (IC, 500 μg.mL^{-1}) for 30 minutes at a shear stress of 2 dyn.cm^{-2} in Bioflux. After washing the microcapillaries, monocytes (10^6 monocytes.mL^{-1} labelled with CMFDA) were perfused for 15 minutes. Monocyte adhesion was quantified at 15 minutes and was expressed as mean ± standard deviation (n=4), *P < 0.05, Mann-Whitney test. As a comparison, level of neutrophil adhesion on IC+PLT is indicated with a dotted line.

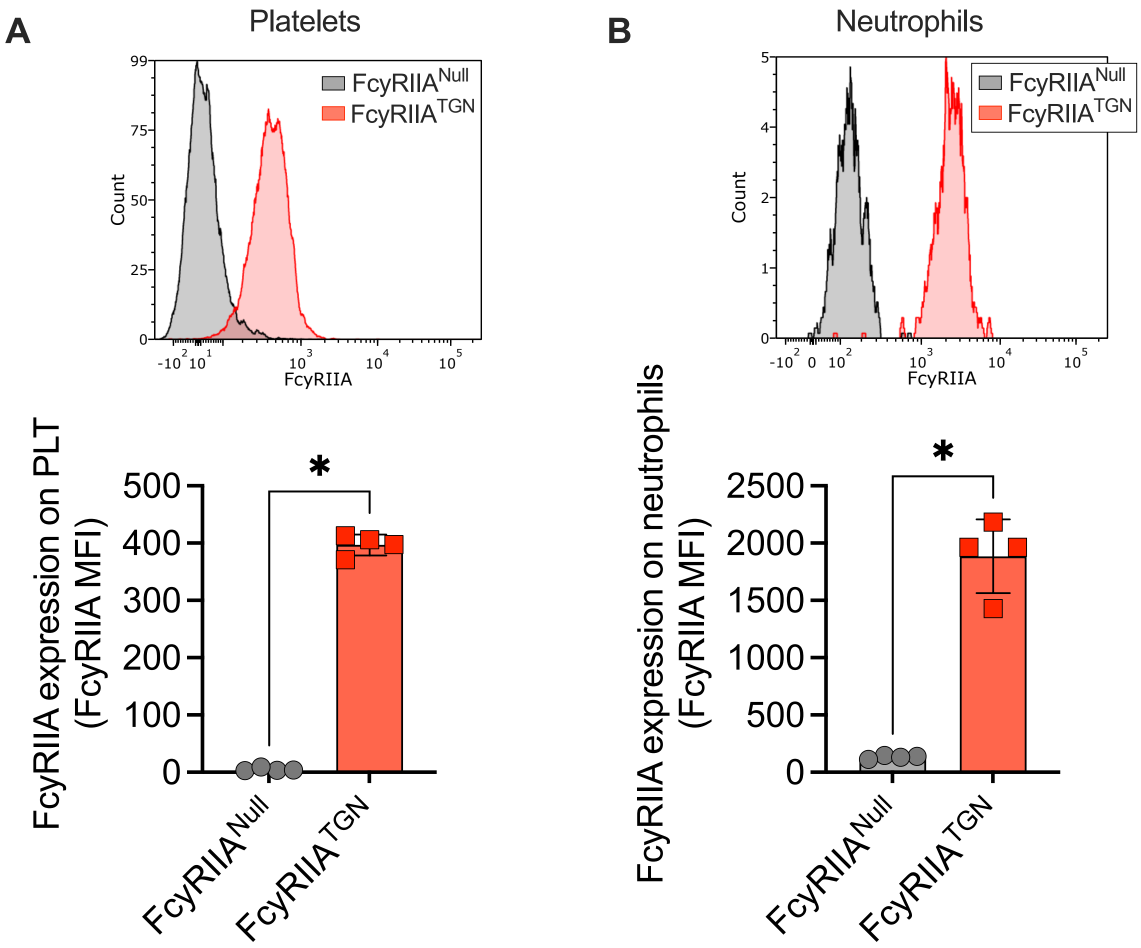

Figure S15: FcyRIIA expression on platelets and neutrophils from FcyRIIA^{Null} or FcyRIIA^{TGN} mice. Level of FcyRIIA expression was evaluated on platelets (A, PLT CD41+ FSC low, grey) and neutrophils (B, CD45+Ly6G+ FCS high, red) in whole blood. Representative histogram (top panels) and Mean Fluorescence Intensity (MFI, bottom panels) are illustrated. Data are expressed as mean ± standard deviation (n=4), *P < 0.05, Mann-Whitney test.

Supplementary videos

Video 1. Human neutrophil adhesion on immune complexes, in absence of platelets, in a microfluidic system. Magnification 200x. Acquisition: one frame every 30 sec for 12 min starting after 3 minutes of shear stress (2 $dyn.cm^{-2}$), timestamp 00 (min) : 00 (sec).

Video 2. Human neutrophil adhesion on immune complexes in presence of platelets, in a microfluidic system. Magnification 200x. Acquisition: one frame every 30 sec for 12 min starting after 3 minutes of shear stress (2 $dyn.cm^{-2}$), timestamp 00 (min) : 00 (sec).

Video 3. Intravital imaging of the joint vasculature (ankle) of a healthy *$FCGR2A^{TGN}$::$Ly6gCre^{+/-}$::$Rosa26\text{-}TdT^{+/-}$::CD41- $YFP^{+/-}$* mouse to visualize platelets (cyan) and neutrophils (red). Magnification 200x. Acquisition: 10 sec in slow motion (0.2x), timestamp 00 (min) : 00 (sec).

Video 4. Intravital imaging of the joint vasculature (ankle) of an arthritic *$FCGR2A^{TGN}$::$Ly6gCre^{+/-}$::$Rosa26\text{-}TdT^{+/-}$::$CD41\text{-}YFP^{+/-}$* mouse to visualize platelets (cyan) and neutrophils (red). Mouse was injected with an isotypic control antibody. Magnification 400x. Acquisition: 10 sec in slow motion (0.2x), timestamp 00 (min) : 00 (sec).

Video 5. Intravital imaging of the joint vasculature (ankle) of an arthritic *$FCGR2A^{TGN}$::$Ly6gCre^{+/-}$::$Rosa26\text{-}TdT^{+/-}$::$CD41\text{-}YFP^{+/-}$* mouse to visualize platelets (cyan) and neutrophils (red). Mouse was injected with an anti-PSGL1 blocking antibody. Magnification 400x. Acquisition: 10 sec in slow motion (0.25x), timestamp 00 (sec) : 00 (sec).

Video 6. Human neutrophil adhesion on immune complexes in presence of platelets, showing pulsing calcium mobilization in a microfluidic system. Magnification 200x. Acquisition: one frame every sec for 3 min, starting one minute after shear stress 2 $dyn.cm^{-2}$, timestamp 00 (min) : 00 (sec).

Table S1: Demographic and clinical characteristics of patients with arthritis.

Variables		Values
Total number of patients		8
Male/Female		3/5
Age of the patients (years; mean ± SD)		69.2 ± 12.6
Duration of the disease (months; mean ± SD)		1.1 ± 2.2
Medication	*NSAIDs*	2 out of 8
	Hydroxychloroquine	4 out of 8
	Methotrexate	4 out of 8
	Prednisone	2 out of 8
RF positive patients (>20 IU/mL)		5 out of 8
Anti-CCP positive patients (>250 IU/mL)		5 out of 8

Abbreviations: NSAIDs (Non-Steroidal Anti-inflammatory Drugs), RF (Rheumatoid factors), CCP (Cyclic citrullinated peptide), IU (international unit).

Table S2: Antibodies used in microfluidic and flow cytometry.

Target	Clone	Fluorochrome	Concentration	cat	Provider
Human PSGL-1	KPL-1	Unconjugated	10 µg.mL^{-1}	#556052	BD Biosciences
Human P-selectin	AK4	Unconjugated	10 µg.mL^{-1}	#551355	BD Biosciences
Human CD11b	ICRF44	Unconjugated	10 µg.mL^{-1}	#555385	BD Biosciences
Human GPIb	HIP1	Unconjugated	10 µg.mL^{-1}	#562255	BD Biosciences
Human FcyRI	10.1	Unconjugated	10 µg.mL^{-1}	#555525	BD Biosciences
Human FcyRIIA	IV.3	Unconjugated	5 µg.mL^{-1}	#60012	StemCell
Human FcyRIIIB	3G8	Unconjugated	10 µg.mL^{-1}	#556617	BD Biosciences
Human CD36	FA6-152	Unconjugated	10 µg.mL^{-1}	#60084	StemCell
Mouse PSGL-1	4RA10	Unconjugated	10 µg.mL^{-1}	#557787	BD Biosciences
Mouse P-selectin	RB40.34	Unconjugated	10 µg.mL^{-1}	#553741	BD Biosciences
Mouse CD11b	M1/70	Unconjugated	10 µg.mL^{-1}	#553307	BD Biosciences
Mouse GPIb	Xia.B2	Unconjugated	10 µg.mL^{-1}	#M043-0	Emfret
Mouse FcyRIII	W20015B	Unconjugated	10 µg.mL^{-1}	#945803	Biolegend
Mouse CD36	MF3	Unconjugated	10 µg.mL^{-1}	#MA5-16833	ThermoFisher scientific
Rat isotype IgG2bk	A95-1	Unconjugated	10 µg.mL^{-1}	#553985	BD Biosciences
Mouse isotype IgG1k	MOPC-21	Unconjugated	10 µg.mL^{-1}	#555746	BD Biosciences
Rat isotype IgG2a	RTK2758	Unconjugated	10 µg.mL^{-1}	#400501	Biolegend
Rat isotype IgG1λ	A110-1	Unconjugated	10 µg.mL^{-1}	#559157	BD Biosciences
Mouse isotype IgG1k	107.3	Unconjugated	10 µg.mL^{-1}	#554721	BD Biosciences
Mouse isotype IgG2b	MPC-11	Unconjugated	5 µg.mL^{-1}	#60072	StemCell
Human activated CD11b	CBRM1/5	APC	4 µg.mL^{-1}	#301410	Biolegend
Human CD41	HIP8	V450	1 µl per test	#561425	BD Biosciences
Human FcyRIIIB	3G8	PE	4 µg.mL^{-1}	#560995	BD Biosciences
Human FcyRIIA	IV.3	FITC	1 µl per test	#100-1574	StemCell
Human P-selectin	AK4	AF488	10 µg.mL^{-1}	#304916	Biolegend
Human P-selectin	AK4	PE	100 µl.mL^{-1}	#555524	BD Biosciences
Human P-selectin	AK4	APC	5 µl per test	#550888	BD Biosciences
Human IgG	F(ab')2 Rabbit polyclonal	AF488	2 µg.mL^{-1}	#309-546-003	Jackson ImmunoResearch
Mouse CD41	MWReg30	BV421	2 µg.mL^{-1}	#747729	BD Biosciences
Mouse P-selectin	RB40.34	FITC	4 µg.mL^{-1}	#553744	BD Biosciences
Mouse αIIbβ3 activated	JON/A	PE	1 µl per test	#M023-2	Emfret
Mouse Ly6G	1A8	APC	4 µg.mL^{-1}	#560599	BD Biosciences
Mouse CD45	30F11	APC-Cy7	4 µg.mL^{-1}	#561037	BD Biosciences
Lactadherin	n/a	FITC	5 µg.mL^{-1}	#Bac-FITC	Prolytix
Annexin V	n/a	FITC	25 µL.mL^{-1}	#556420	BD Biosciences
